# Antimicrobial Efficacy of Green Silver Nanoparticles Synthesized Using *Crataegus monogyna* Extract

**DOI:** 10.3390/biomimetics10110737

**Published:** 2025-11-03

**Authors:** Mihaela Cristina Lite, Roxana Constantinescu, Laura Chirilă, Alina Popescu, Andrei Kuncser, Cosmin Romanițan, Oana Brîncoveanu, Ioana Lăcătușu, Nicoleta Badea

**Affiliations:** 1 National Research and Development Institute for Textiles and Leather—INCDTP, Lucretiu Patrascanu 16, 030508 Bucharest, Romania; cristina.lite@incdtp.ro (M.C.L.); rodica.constantinescu@icpi.ro (R.C.); laura.chirila@incdtp.ro (L.C.); alina.popecu@incdtp.ro (A.P.); 2 National University of Science and Technology POLITEHNICA Bucharest, Faculty of Chemical Engineering and Biotechnologies, 1-7, Polizu Street, 011061 Bucharest, Romania; ioana.lacatusu@upb.ro; 3 National Institute of Materials Physics, Atomistilor 405A, 077125 Bucharest, Romania; andrei.kuncser@infim.ro; 4 National Institute for Research and Development in Microtechnologies, Erou Iancu Nicolae 126A, 077190 Voluntari, Romania; cosmin.romanitan@imt.ro (C.R.); oana.brincoveanu@imt.ro (O.B.)

**Keywords:** *Crataegus monogyna*, green synthesis, silver nanoparticles, antimicrobial textiles

## Abstract

Current demands in the field of functional textiles include the integration of specific characteristics, such as self-cleaning, antimicrobial efficacy and possible wound healing properties. Green synthesis of nanoparticles represents a promising strategy to address these challenges, combining biocompatibility and ecological safety with effective antimicrobial and antioxidant performance. In this study, silver nanoparticles (AgNPs) have been synthesized using different ratios of *Crataegus monogyna* extract: AgNO_3_. Physically stable AgNPs with spherical shape, particle main diameters ranging from 61.9 to 85.4 nm and appropriate polydispersity indices were produced. *Crataegus monogyna* presented high phenolic content (30.58 ± 2.20 mg/g) and strong antioxidant activity (96 ± 1.6 µmol TE/g). The obtained nanoparticles were characterized by TEM, EDX, and XRD analysis. When applied to cotton and wool textiles, the AgNPs adhered uniformly, caused minimal colour change, and exhibited enhanced antimicrobial activity against bacterial and fungal strains compared to other plant-derived AgNPs, with values between 8 and 13.5 mm. The treated textiles demonstrated strong performance against *Staphylococcus aureus* with inhibition zones of 11 ± 0.53 for cotton and 13.5 ± 0.42 for wool. These findings highlight the potential of *Crataegus monogyna*-based AgNPs as effective and fabric-compatible antimicrobial agents.

## 1. Introduction

The scientific progress in the field of functional textiles has reached the point of incorporating self-cleaning properties [1], sensing technologies and electronic components [2], flame retardancy, ballistic protection, wound healing [3], antimicrobial properties [4], and even environmental sustainability contribution, by manufacturing textiles using recycled polymers extracted from plastic [5]. Since the recent SARS-CoV-2 pandemic, greater attention has been paid to global public health, considering that the limitation of spreading infectious diseases is a top priority. Therefore, textile research is focused on developing antimicrobial treatments that can be incorporated into textile fabrics, exhibiting both bactericidal and bacteriostatic properties. The present emphasis is on designing new strategies aimed at enhancing the antimicrobial materials performance, to create a new generation of antimicrobial materials that exhibit greater efficacy and safety [6].

The antimicrobial treatments currently investigated include metal-based nanoparticles, natural polymer-based agents, synthetic polymers, quaternary ammonium compounds, natural dyes, and microencapsulation of essential oils [7]. The advantages of these treatments consist of broad-spectrum efficacy (bacteria, fungi, viruses) and durable performance [8]. Natural polymers such as chitosan and its derivatives are biocompatible, biodegradable, film-forming, and highly effective against Gram-positive bacteria, but they are less effective against Gram-negative bacteria and require crosslinking for wash durability [9,10]. Polyhexamethylene biguanide (PHMB), quaternary ammonium compounds (QACs) and triclosan possess strong bactericidal and virucidal activity, and they are widely used in healthcare textiles. However, they may cause skin irritation and represent a potential environmental risk from persistent residues [11].

The advantages of using metal-based nanoparticles are represented by their broad-spectrum antimicrobial efficacy, reduced risk of antibiotic resistance, durability, and biocompatibility [12,13,14,15]. The challenges and risks encountered might comprise the potential cytotoxicity, environmental impact, breathability alterations, photoactivation requirements (e.g., TiO_2_), ions leaching, cost and regulatory [16,17,18].

Due to rapid nanotechnology advancements, the range of possibilities has become wider. Nanoparticles (NPs) possess functionalities that have propelled them to be investigated for various applications, such as sensors [19], antimicrobial biomaterials [20], catalysis [21], drug delivery [22], etc. The green approaches in NPs production represent one solution to reduce the costs and to avoid the use of hazardous reagents, while maintaining the efficiency [23,24]. The environmentally friendly, rapid, and straightforward method of plant-mediated green synthesis of metal NPs involves the valorization of bioactive polyphenols from herbals, such as phenolic acids and flavonoids, which serve as reducing agents, coating agents or stabilizers. This process leads to the obtaining of NPs that possess the desired size, shape, and properties [25]. In a related study, Shivanjali et al. underlined the physical behaviour of AgNPs obtained through chemical coprecipitation and an eco-friendly method that used the Cassia fistula L extract [26]. The average particle size for the NPs produced chemically was 43.6 nm, while the eco-friendly method assured a mean size of 58.5 nm. The antioxidant and anti-inflammatory properties of the AgNP synthesized using the ecological method were found to be superior, exhibiting an antioxidant activity of 84.48% compared to 75.87% for those synthesized chemically, with IC50 values of 30.19 μg/mL versus 34.14 μg/mL. In another study, green AgNPs obtained using *D. stramonium* extract exhibited a narrow size distribution, spherical shape, and demonstrated high levels of antioxidant and DNA cleavage activities. The chemically synthesized AgNPs had a smaller average size, no antioxidant activity, while having reduced antibacterial activities [27].

A. L. dos Santos et al. investigated the antimicrobial potential of silver nanoparticles applied to cotton gauze, using beetroot extract (*Beta vulgaris*). Even at low concentrations, AgNPs dispersions led to a 50% to 90% inhibition of microbial growth of bacterial strains of *E. coli*, *S. epidermidis*, *S. aureus* and the fungal strain of *C. albicans* [28]. In a similar study, the synthesis of AgNPs was performed using red sandalwood (*Pterocarpus santalinus*) flowers. Textile fibres treated with AgNP suspension exhibited a clearly defined zone of inhibition. This zone was more extensive in the case of the sample subjected to ultrasound, demonstrating an increase in the antibacterial effect due to the significant rise in the contact surface of AgNPs with pathogens, by ensuring a uniform distribution of the nanoparticle layer [29]. Fatma et al. examined the antimicrobial effect of AgNPs on linen textile samples using a complex polysaccharide secreted by the bacterial strain *Bacillus subtilis*. Tests on bacterial strains of *E. coli* and *S. aureus* showed that the treated samples retained their antibacterial properties even after 20 washing cycles [30]. The synthesis route of AgNPs using an aqueous extract of guava leaf (*Psidium guajava* Linn.) has been reported by Filho et al. Antimicrobial and cytotoxicity tests were conducted on treated cotton fabric and showed minimal to no cytotoxicity. Further research involving additional cell lines, various cytotoxicity assays, and in vivo studies is essential to confirm the safety of AgNPs-functionalized textiles. Both the AgNPs solution and the treated fabric demonstrated antimicrobial activity against standard reference strains and clinically relevant, drug-resistant bacteria, including *Staphylococcus aureus*, *Enterococcus faecalis*, *Klebsiella pneumoniae*, *Escherichia coli*, *Pseudomonas aeruginosa*, and *Acinetobacter baumannii*. The only exception was an ESBL-producing *E. coli* strain, which exhibited resistance to AgNPs. Atomic force microscopy analysis revealed that the antimicrobial activity of AgNPs is mainly due to structural damage inflicted on the bacterial cell wall [31]. The antimicrobial potential of cotton fabrics with silver NPs synthesized using *Cichorium intybus* extract has revealed the formation of a clear zone of inhibition when testing the extract alone against microbial and fungal strains. The size of the inhibition zone was higher for the AgNPs-treated cotton fabric [32].

The research group of T. Ahmed synthesized AgNPs using *Calendula arvensis* extract at a controlled temperature and then incorporated them into nonwoven fabric through both padding and in situ methods. The in situ application of AgNPs resulted in up to 99% antibacterial effectiveness on the nonwoven surface. Additionally, fabrics treated via the in situ method outperformed those treated by the padding method in terms of both particle filtration efficiency (PFE) and bacterial filtration efficiency (BFE) [33].

Treatment of silk with silver-green NPs obtained from *Premna serratifolia* L. resulted in the production of a material with superior antibacterial properties of over 99.78% against *S. aureus* and over 99.99% against *Escherichia coli*, which persist after 5–10 washing cycles [34]. Typically, when applied to textiles, AgNPs were reported to provide antimicrobial efficacy even after 20 washing cycles [35,36,37,38].

Environmentally friendly AgNPs obtained by implications of various herbals, e.g., madder dyes [39], natural dyes extracted from neem [40], and hibiscus flower extract [41] were used simultaneously to dye fabrics and to obtain multifunctional textiles that protect against pathogenic microorganisms and UV radiation. In this context, *Crataegus monogyna* represents a viable option for the synthesis of AgNPs. *Crataegus monogyna* extract is a medicinal plant widely distributed in America, Europe, and Asia, with a rich content of polyphenols, particularly flavonoid glycosides and proanthocyanidins, used in the treatment of cardiovascular diseases (hypertension, arrhythmia), hyperlipidemia, digestive disorders, and cancer [42]. This plant has been used to obtain NPs with medical applications, presenting antibacterial and significant anticancer effects against breast and gastric cancer cell lines [43,44].

The use of natural, herbal-based principles for the eco-friendly synthesis of AgNPs aligns with the goals of biomimetic research. Biomimetics inspires “green chemistry” approaches, where herbal extracts act as non-toxic reducing and stabilizing agents, mimicking the gentle processes found in living organisms. This eco-friendly synthesis of AgNPs successfully achieved by utilizing *Crataegus monogyna* extract is monitored by optimizing the ratio of AgNO_3_ to extract, size, and related physical stability. Additionally, our investigation quantifies the required amount of polyphenols from *Crataegus monogyna* extract for the production of AgNPs and demonstrates the ability of the resulting silver nanoparticles to scavenge both short-lived and long-lived radicals. Another novelty aspect concerns the examination of the chromatic parameters determined for wool and cotton samples and the correlation with the antimicrobial effects of AgNPs derived from *Crataegus monogyna* extract on various textile materials. To our knowledge, this is the first work reporting the use of silver nanoparticles obtained from aqueous *Crataegus monogyna* extract in textiles, demonstrating notable antioxidant activity on harmful reactive species and antimicrobial effects against Gram-positive and Gram-negative bacteria, as well as the fungal strain *Penicillium hirsutum*.

## 2. Materials and Methods

### 2.1. Materials

Silver nitrate was purchased from Anal-R NORMAPUR, and *Crataegus monogyna* dry plant from Fares (Oradea, Romania). A mixture of phosphomolybdate and phosphotungstate (Folin-Ciocâlteu reactive), 2,2-azinobis-(3-ethylbenzthiazoline-6-sulfonic acid) (ABTS), anhydrous sodium carbonate, Trolox (6-hydroxy-2,5,7,8-tetramethylchroman-2-carboxylic acid), potassium persulfate, gallic acid, and sodium chloride were purchased from Sigma Aldrich Chemie GmbH (Munich, Germany). Tris (hydroxymethylaminomethane base) and 5-amino-2,3-dihydro-phthalazine-1, 4-dione were bought from Merck (Darmstadt, Germany). The culture media used for antimicrobial activity were Casein Soya Bean Digest (SCDLP), Casein Soya Bean (TSA), Digest Agar (DA), Enumeration Agar (EA), Tryptic Soy Broth (TSB), and Nutrient Broth (NB).

### 2.2. Synthesis and Characterization of Green Silver Nanoparticles

#### 2.2.1. Synthesis and Optimization Strategies of AgNPs

The aqueous extract of *Crataegus monogyna* used for the ecological synthesis of AgNPs was prepared by infusing the plant for 30 min and then filtering. The tested concentrations of herbal extract were 3 g/100 mL and 1 g/100 mL. The chemical composition of the extract was determined using HR-mass spectrometry with a Fourier transform ion cyclotron resonance spectrometer (FT-ICR, model SolariX XR 15T, Bruker Daltonics, Bremen, Germany). Conditions for FT-ICR analysis with positive ESI ionization: 310 µL/h (the flow rate of sample), N_2_ at 1.2 bar, 180 °C.

AgNPs were synthesized by mixing different proportions of silver precursor (1 mM AgNO_3_) and plant extract in transparent containers. The incubation of reaction mixtures was performed in a sunlight simulation chamber. The incubation time was 24 h, at room temperature; subsequently, the reaction mixtures were transferred to brown containers. Using 3 g/100 mL extract the following ratios (*v*/*v*) were prepared: 9:1, 5:1, 3:1, 1:1, 1:3, 1:5, 1:9. With the extract of 1 g/100 mL, the ratios (*v*/*v*) prepared were 1:1, 1:2, 1:3, 1:4, 1:5, 1:9, 1:11, 1:15.

Thus, the green synthesis was carried out where Ag^+^ ions were reduced and the resulting AgNPs were captured and stabilized by the phyto-components contained in the aqueous extract of *Crataegus monogyna*. By monitoring the characteristic absorptions (UV-Vis spectroscopy) and by evaluating the size and physical stability, the synthesis was optimized. The polyphenol consumption was determined using the Folin–Ciocâlteu method, and the antioxidant activity was evaluated using chemiluminescence and TEAC methods. The characterization was completed using transmission electron microscopy (TEM) together with energy-dispersive X-ray spectroscopy (EDX) and X-ray diffraction (XRD) techniques. AgNP dispersions were applied to natural textile samples (cotton and wool), and the treated samples were further characterized to quantify the treatment efficiency by evaluating the chromatic parameters and the antimicrobial effect against bacterial strains of *Escherichia coli*, *Staphylococcus aureus*, *Bacillus subtilis*, as well as the fungal strain of *Penicillium hirsutum*.

#### 2.2.2. The Physico-Chemical and Morphological Characterization of Silver Nanoparticles

Spectral characteristics of the AgNPs aqueous dispersions prepared in various herbal extract and silver precursor ratios were evaluated using a Lambda 950 instrument from Perkin Elmer (Waltham, MA, USA). UV-VIS analysis was performed in the spectral range 200–700 nm. The electron density oscillation on the particle surface, induced by their nanosize, absorbs electromagnetic radiation. This oscillation recognised as surface plasmon resonance (SPR) indicates the silver nanoparticles formation [45].

The average size (Z_av_) and the polydispersity index (PDI) of the AgNPs were determined by dynamic light scattering (DLS) using a Zetasizer NanoZS (Malvern Instruments Inc., Worcestershire, UK). The polydispersity index represents the distribution of the particle populations as a function of size. Three measurements (at 25 °C) were achieved for each sample, and the mean values were reported.

The physical stability of the AgNPs was reported in terms of zeta potential measurements. The zeta potential (ξ) was measured in a capillary cell (using the Helmholtz-Smoluchowski equation) by converting the particle electrophoretic mobility. An amount of 300 µL of AgNPs aqueous dispersion was added to 20 mL of distilled water. The zeta potential values were determined by applying an electric field and by using the Zetasizer Nano ZS (Malvern Instruments Inc., Worcestershire, UK).

The morphological characterization was done by high-resolution TEM (Cs probe-corrected JEM ARM 200F transmission electron microscope, JEOL Ltd., Tokyo, Japan). The crystallinity was followed by X-ray diffraction (9 kW Rigaku SmartLab equipment, equipped with a Cu Kα_1_ source, λ = 0.154 nm (Tokyo, Japan). The measurements were performed in a grazing incidence mode. The incidence angle (ω) was fixed at 0.5, and 2θ varied from 35 to 80°.

#### 2.2.3. The In Vitro Evaluation of Antioxidant Activity

Capacity of AgNPs to scavenge the dangerous oxygen free radicals and cationic radicals was determined using the chemiluminescence (CL) assay and the TEAC method. The analyses were performed comparatively between AgNP dispersions and plant extract. The short-life oxygen-free radicals (ROS) were generated for chemiluminescence measurements (Turner Design TD 20/20 USA Chemiluminometer) by luminol (0.01 mM), TRIS-HCl buffer solution (pH 8.6) and hydrogen peroxide (0.01 mM). This mixture was used as a blank solution [46]. The antioxidant activity (AA%) was calculated by Equation (1):

%AA = (I_0_ − I_s_) × 100/I_s_ (1)

where I_0_ = the maximum CL intensity for the reference; I_s_ = the maximum CL intensity for the tested samples.

The antioxidant activity assessed by the TEAC method involved the spectral monitoring of the long-lived radical ABTS^●+^, which is generated by the reaction between 7 mM solution of 2,2-azinobis-(3-ethylbenzthiazoline)-6-sulfonic acid solution and 2.45 mM solution of K_2_S_2_O_8_. The normalization of ABTS^●+^ solution was made after 16 h, at 734 nm in order to achieve an absorbance value of 0.70 ± 0.02 (UV-VIS-NIR V670 spectrophotometer, Jasco, Tokyo, Japan). The calibration curve was build using Trolox standard solutions with a concentration range of 0 ÷ 60 μM (R^2^ = 0.9989). The ABTS^●+^ inhibition value was calculated with Equation (2):

%Inh ABTS^●+^ = (A_0_ − A_s_) × 100/A_0_ (2)

where A_0_ is the absorbance of the control (3 mL previously normalized ABTS^●+^ solution and 2 mL distilled water) and A_s_ is the absorbance of the samples (3 mL previously normalized ABTS^●+^ solution, 0.5 mL diluted AgNPs dispersion/extract and 1.5 mL distilled water). After measuring each sample three times, the antioxidant capacity was reported as Trolox equivalent.

#### 2.2.4. Polyphenol Determination for Entrapment Efficiency

The phenolics amount for both herbal extract and silver nanoparticles was determined using the Folin–Ciocâlteu method, in accordance with ISO 14502-1:2005 [47] and was reported in gallic acid equivalent (GAE). A calibration curve with a concentration range of 0–60 µg/mL and R^2^ = 0.9997 was used. An amount of 0.5 mL AgNP dispersion and herbal extract was mixed with 4.5 mL of 7.5% (m/m) anhydrous Na_2_CO_3_ solution and 5 mL of 10% (*v*/*v*) Folin–Ciocâlteu reactive. After incubating the samples in a dark place for one hour at room temperature, their absorbance was recorded in triplicate at λ = 765 nm. To calculate the average polyphenol consumption, the polyphenol content of the AgNP dispersion was subtracted from the phenolic content of the extract solutions.

### 2.3. Textiles Treatment and Characterization

The antimicrobial action was tested on cotton and wool samples, after several textile fabrics (10 × 10 cm) were immersed in the AgNP dispersion. The samples were left to dry overnight. The morphological, chromatic and antimicrobial properties of the treated fabrics were evaluated.

For morphological changes, the scanning electron microscopy (SEM, FEI Quanta 200 instrument, Everhart–Thornley (ET) detector, ThermoFisher Scientific, Waltham, MA, USA) was used. The accelerating voltage was 15 kV, and the characterization was performed in low vacuum mode. The nature of the deposited AgNPs was confirmed by X-ray dispersive spectroscopy (EDX), by using an X-ray detector (from EDAX AMETEK, Berwyn, PA, USA) along with an electronic microscope.

To determine the chromatic parameters, a Datacolor instrument (Datacolor, Inc., Lucerne, Switzerland) was used. In the CIE L*a*b* system of colours, the L* parameter correlates with the luminosity of the samples, while a* and b* indicate the colour of the samples. The values of the L* are in the range 0 ÷ 100 (0 = black colour; 100 = white colour). The values of the a* and b* parameters are situated between 100 and +100. After determining the parameters for the untreated textile fabric and for the treated samples, a total colour change can be calculated using the formula [48]:

ΔE* = [(ΔL*)^2^ + (Δa*)^2^ + (Δb*)^2^]^1/2^ (3)

The antimicrobial tests of the AgNPs dispersions were carried out against bacterial such as *Escherichia coli*, *Staphylococcus aureus*, and *Bacillus subtilis* and *Penicillium hirsutum* fungal strains.

The antibacterial tests were achieved according to ISO 20743: 2013 [49]. The colony-forming units (CFUs) were counted after a direct inoculation of the bacteriological inoculum onto the treated samples and an incubation time of 24 h. Hence, the bactericidal percentage, R (%), was calculated using Equation (4):

R (%) = (CFU_control_ − CFU_sample_) × 100/CFU_control_ (4)

where CFU_control_ = the number of colony-forming units of the control sample and CFU_sample_ = the number of colony-forming units of the treated samples.

Furthermore, the agar diffusion method was used to determine the degree of bacterial and fungal inhibition, respectively [50,51]. For this analysis, *Escherichia coli* ATCC 10536, *Staphylococcus aureus* ATCC 6538, *Bacillus subtilis* ATCC 6633 bacterial strains, and *Penicillium hirsutum* ATCC 52323 fungal strain were tested. Each strain was spread on Petri dishes surface. The textiles (with a diameter of 10 mm) were placed on the nutrient medium surface, in the Petri dishes centre. After an incubation time of 24 h and 37 °C, the antimicrobial action was recognized by the identification of a clear inhibition zone (IZ) around the sample. This inhibition area was calculated using Formula (5):

IZ = (D − d)/2(5)

where D = the textile sample diameter plus the inhibition zone (mm) and d = the textile sample diameter (mm).

### 2.4. Statistical Analysis

All measurements were taken in triplicate at 25 °C and were reported as mean value ± standard deviation. Significant differences among the experimental groups were statistically analysed using one-way analysis of variance ANOVA. Statistical significance was considered at *p* < 0.05.

## 3. Results and Discussion

### 3.1. Compositional Characterization of Crataegus Monogyna Extract by FT-ICR MS

**Figure 1:**
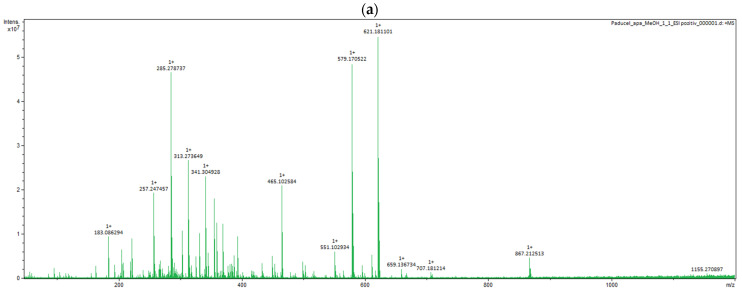

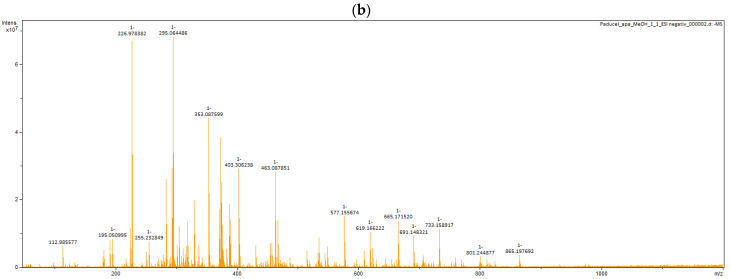
Mass spectra obtained by (**a**) positive and (**b**) negative ionization for *Crataegus monogyna* extract.

The characterization carried out by FT-ICR MS analysis of the aqueous extract (Figure 1) revealed the presence of: epicatechin (291.0861 m/z/ESI+, 289.0716 m/z/ESI−), quercetin (m/z 303.0498) and quercetin derivatives such as: quercetin 3-O rhamnoside-7-O-glucoside (611.1600 m/z/ESI+, 609.1455 m/z/ESI−), quercetin-3-D-xyloside (433.0775 m/z/ESI−). Also identified, by both positive and negative ionization, were: chlorogenic acid (355.1022 m/z/ESI+, 353.0876 m/z/ESI−) and 4,5-dicaffeoylquinic acid (517.1332 m/z/ESI+, 515.1191 m/z/ESI−), luteolin-7-O-glucoside (449.1077 m/z/ESI+, 447.0928 m/z/ESI−) and apigenin 6-C-glucoside/Isovitexin (m/z 433.1129 m/z/ESI+, 431.0980 m/z/ESI−).

The m/z data determined experimentally are consistent with those calculated, the theoretical m/z mass (Table 1). MS spectra obtained for ESI+ and ESI− are monoisotopic and are included in the Supplementary Materials (Figures S1–S14).

Ruiz-Rodriguez et al. (2013) identified in *Crataegus monogyna* extract from Spain: gallic acid, chlorogenic acid, picatechin, quercetin 3,4-diglucoside, quercetin 3,7,4-triglucoside and cyanidin 3-galactoside [52]. Also, Cosmulescu et al. (2017), using the HPLC-PDA method, quantified the following compounds in hawthorn extract from Romania: gallic acid, caffeic acid, syringic acid, ellagic acid, chlorogenic acid, vanillic acid, epicatechin, coumaric acid, ferulic acid, salicylic acid, sinapic acid, rutin, myricetinic acid, trans-cinnamic acid and quercetin [53].

**Figure 2:**
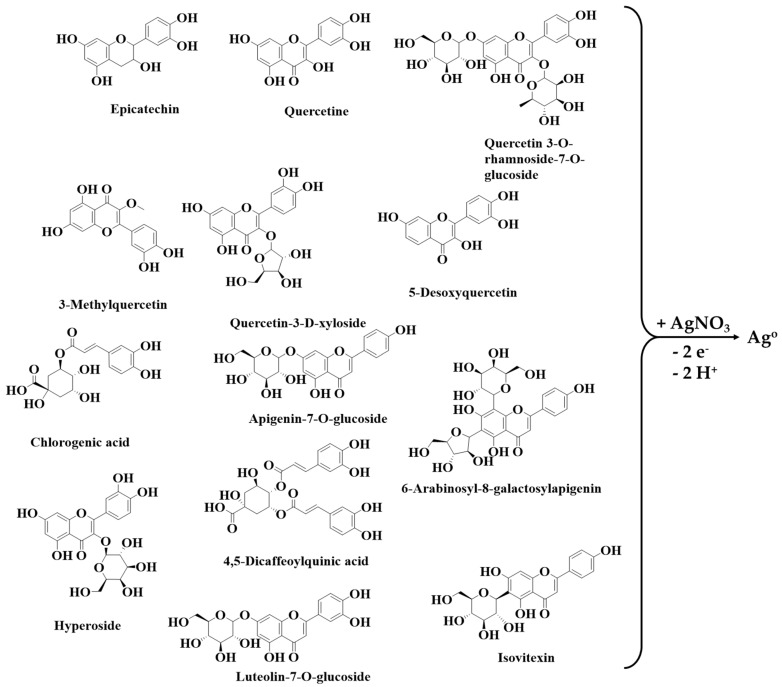
Mechanism of AgNPs green synthesis using *Crataegus monogyna* plant extract.

The phenolic compounds identified in *Crataegus monogyna* extract function as reducing agents for silver ions, leading to the synthesis of silver nanoparticles (Figure 2).

### 3.2. UV-VIS Spectral Characterization of AgNPs Dispersions

**Figure 3:**
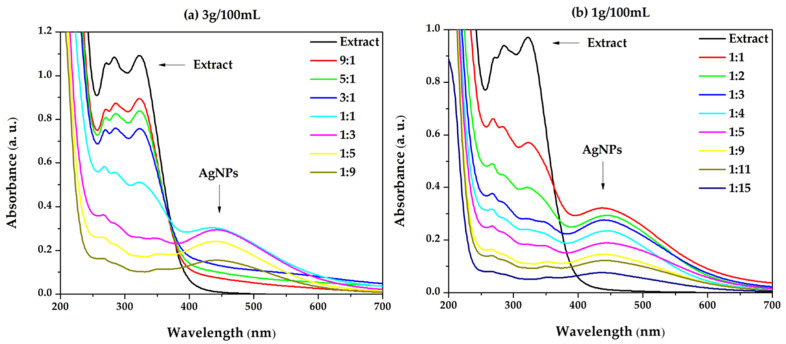
Overlaid UV-VIS absorption spectra of AgNPs dispersions at various ratio between herbal extract concentrations and AgNO_3_ (*v*/*v*): (**a**) 3 g/100 mL; (**b**) 1 g/100 mL.

The synthesis of Ag nanoparticles was carried out using two different extract concentrations: 1 g/100 mL and 3 g/100 mL. Figure 3 displays the UV-Vis spectra for both concentrations, indicating that the formation of AgNPs is affected by both the extract concentration and AgNO_3_. Consequently, a relationship can be observed between the various extract: Ag^+^ ion precursor ratios: increasing the silver ion concentration resulted in a decrease in the absorption maximum, and higher extract concentrations inhibited the formation of silver nanoparticles, as demonstrated by the absence of the SPR band. The absorption maximum of the SPR band occurs at an extract/AgNO_3_ ratio of 1:1.

The shape, intensity, and position of the SPR band are influenced by the particle size and distribution. Thus, a narrow band indicates the formation of a homogeneous dispersion of nanoparticles, while a broad band suggests the formation of agglomerations leading to the appearance of several particle populations. At the same time, the shift of the SPR band towards shorter wavelengths is an indicator of the formation of small-sized nanoparticles [54].

In the present case, the shift of the SPR band with the absorption maximum at λ = 439 nm was minimal, suggesting that the AgNPs size was maintained in a relatively small range, unlike the dispersions obtained with the *Stellaria media* extract [55]. On the other hand, the width of the SPR bands changes as the silver precursor concentration increases, suggesting the formation of agglomerations. The same behaviour of the decrease in intensity of the SPR band with increasing AgNO_3_ ratio was also obtained by Nadzir, who used aqueous extract of *Gynura procumbens* in the synthesis of AgNPs. A possible explanation is related to the competition that can occur with the presence of a large amount of silver ions compared to the available biological material, and which can lead to the formation of fewer nanoparticles that present electronic oscillations, and therefore less intense SPR bands [56]. C. Tănase reported the same behaviour following his study on the biosynthesis of AgNPs using aqueous extract of *Picea abies* L. [57].

### 3.3. Dimensional and Physical Stability Evaluation of Nanoparticles

**Figure 4:**
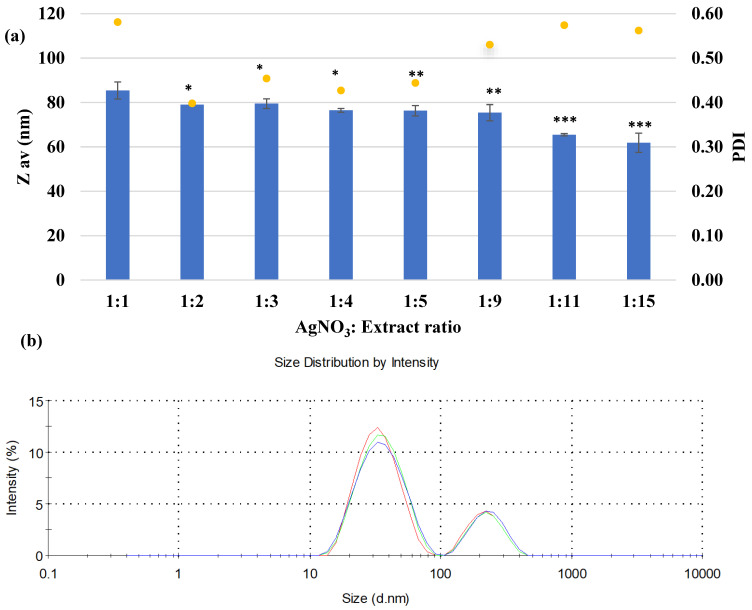
Average size (Z_ave_, nm) and polydispersity index (PDI) of AgNPs, determined by DLS technique (**a**) Particle size distribution for 1:3 ratio (**b**). Experiments were achieved in triplicate, and the data are expressed as mean ± SD, for a ratio of 1:1 compared to other groups. * *p* < 0.05 ** *p* < 0.005 *** *p* < 0.0005.

The average size of the obtained AgNPs (Figure 4a), evaluated by the DLS technique, is in the range of 61.9–85.4 nm, being slightly decreased, from the ratio 1:1 to the ratio 1:15. Regarding the homogeneity of the samples, they follow an irregular trend, with polydispersity index values in the range 0.398–0.581, the minimum value being recorded for the ratio 1:2. Figure 4b reveals that the particles size distribution consists of two populations from which the biggest one is in the nano range.

The sizes of the silver particles obtained with the *Crataegus monogyna* extract were maintained in a relatively narrow range, with a difference of approximately 20 nm between the minimum and maximum values, suggesting a high stability, conferred by the phytocompound content of this extract. Mohammad Ali reported obtaining a similar range of average sizes, ranging from 40 to 80 nm, when using the aqueous extract of *Artemisia absinthium* in the synthesis of AgNPs. As in the present case, the average particle size decreased with increasing silver ion content [58]. Regarding the homogeneity of NPs, quantified by the value of the polydispersity index, PDI, it is known that a value close to 1 is associated with particle aggregation, the population is more polydisperse, while PdI values in the range of 0.1 ÷ 0.3 suggest the absence of agglomerates and the obtaining of uniformly distributed nanoparticles (when the value tends towards 0.1) [59]. The PDI index may depend on the extract: AgNO_3_ ratio and the nature of the extract. While some studies have reported a decrease in the PDI index with increasing AgNO_3_ content [60], others have reported an irregular variation [61], and some studies have observed an increase in the PDI index with increasing AgNO_3_ concentration.

**Figure 5:**
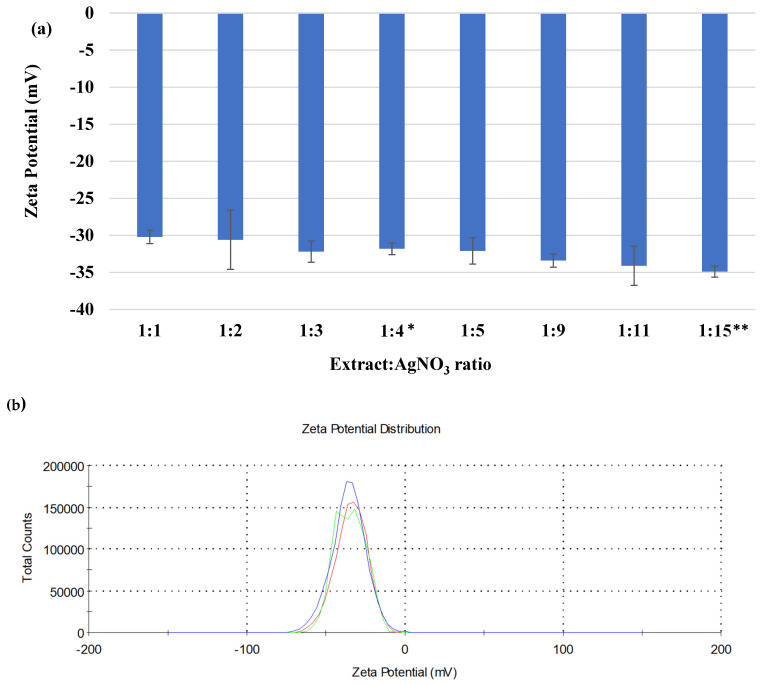
(**a**) Physical stability of AgNPs-P dispersions, expressed by zeta potential (**b**) Zeta potential distribution of a 1:3 ratio. All experiments were performed in triplicate. * *p* < 0.05; ** *p* < 0.005; Data are expressed as mean ± SD, n = 3, Ratio 1:1 vs. other groups.

Using the *Crataegus monogyna* extract in the synthesis of AgNPs, dispersions with high stability were obtained, with absolute values of zeta potential in a slight increase, from −30 mV for the 1:1 ratio to −35 mV for the 1:15 ratio (Figure 5a). The zeta potential values of AgNPs dispersions revealed the further presence of negatively charged polyphenolic components on the nanoparticle surface [62]. Figure 5b reveals the zeta potential distribution.

As in the case of particle size, the range of zeta potential values is narrow (with a difference of 5 mV between the minimum and maximum value). This aspect confirms the high stability conferred by the phytochemical compounds in the *Crataegus monogyna* extract. The trend of a slight increase in stability with increasing silver ion content correlates with the decrease in the average particle size and the increase in the polydispersity index. The dependence of the zeta potential on the extract to AgNO_3_ ratio depends on the nature of the extract. When *Laminaria japonica* extract or *Imperata cylindrical* extract was used in the synthesis of AgNPs, the absolute value of the zeta potential decreased with increasing silver ion content [63]. On the other hand, when *Lantana camara* leaf extract was used, the effect was to increase the stability of the AgNPs dispersions. High negative values illustrate the repulsion between particles and, therefore, obtain good stability of the silver nanoparticles, avoiding their agglomeration due to the repulsive forces between them [64].

### 3.4. Determination of Antioxidant Potential and Phenolic Content

**Figure 6:**
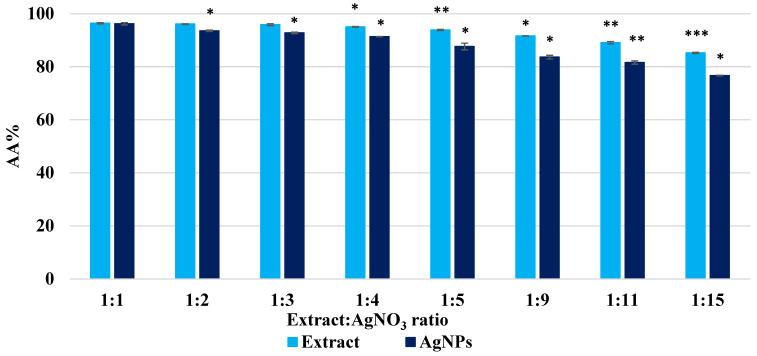
Antioxidant activity of *Crataegus monogyna* extract solutions and AgNPs dispersions, evaluated by the chemiluminescence method. All experiments were performed in triplicate. Data are expressed as mean ± SD, n = 3, ratio 1:1 vs. other groups. * *p* < 0.05; ** *p* < 0.005; *** *p* < 0.0005.

The antioxidant potential of AgNPs compared to the plant extract was quantified by the chemiluminescence method and the ABTS method. The antioxidant efficacy revealed a short-lived radical scavenging capacity of oxygen radical species (ROS) with percents of 76–96% for extract solutions and dispersions of AgNPs (Figure 6). Even if it seems that the antioxidant activity of AgNPs is lower than that of the extract, this decrease is smaller compared to other extracts studied [55,65]. Moreover, the extract solutions and AgNPs dispersions were diluted 10 times to obtain comparable values; hence, the antioxidant activity quantified by chemiluminescence was much higher compared to other extracts.

**Figure 7:**
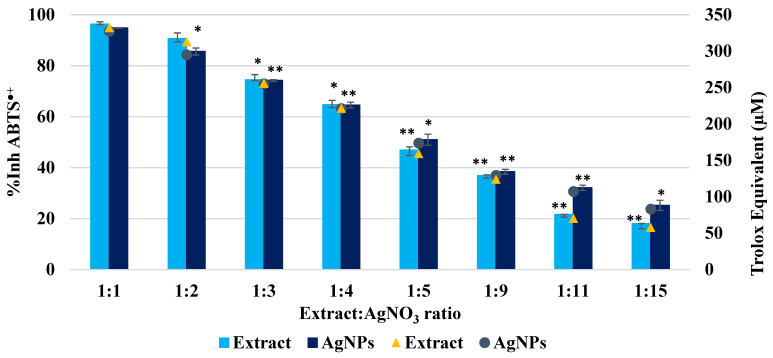
Antioxidant activity of *Crataegus monogyna* extract solutions and AgNPs dispersions, evaluated by the TEAC method. All experiments were performed in triplicate. * *p* < 0.05; ** *p* < 0.005; Data are expressed as mean ± SD, n = 3, Ratio 1:1 vs. other groups.

The long-lived ABTS^●+^ radical scavenging capacity of AgNPs varies in the range of 25–95% and depends on the extract to AgNO_3_ ratio. Thus, up to the ratio of 1:4, it is found that there are no significant differences between the extract samples and the AgNPs dispersions. In contrast, for the following ratios, the activity of AgNPs is superior to the extract, for example, at the ratios of 1:11 and 1:15. The antioxidant activity of the extract, expressed in Trolox equivalent, is 96 ± 1.6 µmol/g of dry plant. Figure 7 presents the antioxidant activity of the AgNPs dispersions and extracts, expressed in Trolox equivalents.

Unlike other previously studied extracts, for which the Trolox equivalent increased when AgNPs were present in the system [55,65], in the present case, it is lower for AgNPs dispersions, compared to extract solutions, for the ratios 1:1 and 1:2, while for the ratios 1:3 and 1:4, it remains constant at 250 and, respectively, 220 μM. For the rest of the ratios, an increase in antioxidant activity is observed when AgNPs are present. This behaviour is due to the fact that, when the *Crataegus monogyna* extract content is higher, it is mainly responsible for capturing long-lived radicals. On the other hand, when the extract content is lower, the increase in antioxidant activity is attributed to the nanosize of AgNPs. This behaviour indicates a strong antioxidant capacity of the *Crataegus monogyna* extract. The mechanisms driving the enhanced antimicrobial activity compared to other herbal-derived AgNPs arise from the synergistic effect of the antioxidant effect of both AgNPs and *Crataegus monogyna* extract.

**Figure 8:**
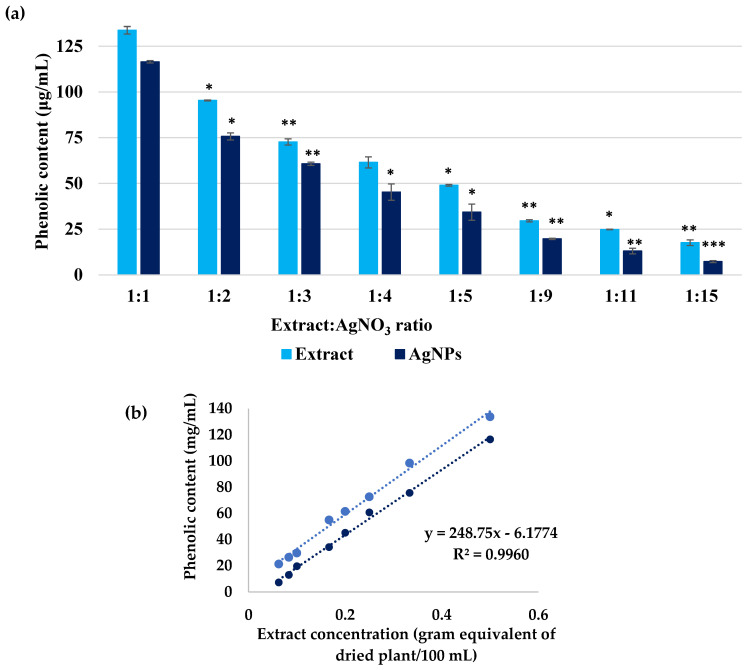
(**a**) Phenolic content of *Crataegus monogyna* extract solutions and AgNPs dispersions, evaluated by the Folin–Ciocâlteu method. (**b**) Dependence of phenolic content on extract concentration. All experiments were performed in triplicate. * *p* < 0.05; ** *p* < 0.005; *** *p* < 0.0005; Data are expressed as mean ± SD, n = 3, Ratio 1:1 vs. other groups.

The phenolic content, determined by the Folin-Ciocâlteu method (Figure 8), was 30.58 ± 2.20 mg GAE equivalent/g dry plant, and the amount of polyphenols used in the synthesis of AgNPs was 1.58 mg, representing 5% of the total polyphenol content.

The extract of *Crataegus monogyna* presented a relatively high phenolic content and, at the same time, a strong antioxidant activity. The polyphenol content of this extract is comparable to that determined for the plants *Rhodiola rosea* and *Ziziphus mauritiana* [66,67]. Also, Kosti’c et al. [68] found that the total amount of phenolic substances in the hawthorn samples obtained is 2.12–30.63 mg GAE g^−1^. Regarding the antioxidant activity determined by the TEAC method, the same behaviour of increasing the Trolox equivalent following the synthesis of AgNPs was observed by Csakvari and collaborators when they used an extract of *Cannabis sativa* leaves [69]. Similar results were also reported when the extract of *Salvia aethiopis* was used in the synthesis of AgNPs [70].

### 3.5. Morphology and Crystallinity Characterization of AgNPs Obtained with Crataegus Monogyna Extract

**Figure 9:**
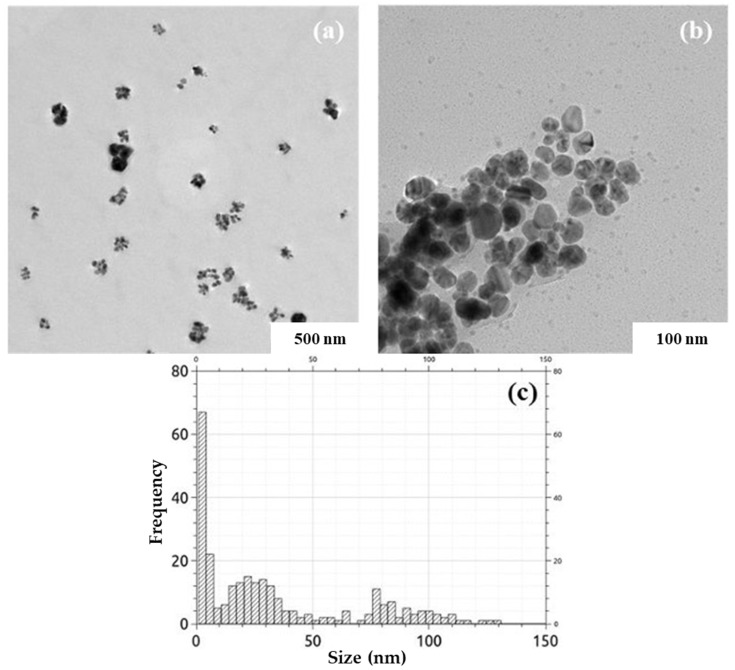
TEM images of AgNPs 1:3: (**a**) 500 nm scale; (**b**) 100 nm scale; (**c**) size distribution of dominant morphologies (quasi-spherical, <100 nm).

**Figure 10:**
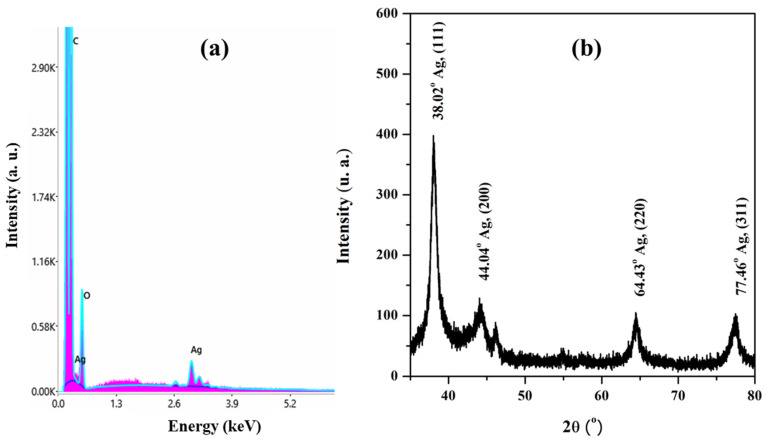
(**a**) EDX spectrum and (**b**) XRD diffractogram of AgNPs.

The ratio of extract to silver ions of 1:3 (extract concentration of 1 g/100 mL) was selected as the optimal one, presenting a high physical stability of AgNPs in dispersion of (ξ = −32.20 ± 1.44 mV), an average size of AgNPs of 79.47 ± 2.18 nm. The TEM images of AgNPs synthesized using *Crataegus monogyna* (Figure 9) at this ratio were similar to those obtained with *Stellaria media* extract [55], showing only quasi-spherical silver nanoparticle systems of 30 nm and 100 nm, respectively, which tend to agglomerate into micrometric sized clusters. Similar images were obtained for AgNPs synthesized with *Moringa oleifera* extract [64]. The EDX spectrum and XRD diffractogram (Figure 10) confirmed the nature and crystallinity of silver [71].

### 3.6. Morphology of Textile Fibres Treated with AgNPs Dispersion Based on Crataegus Monogyna Extract

**Figure 11:**
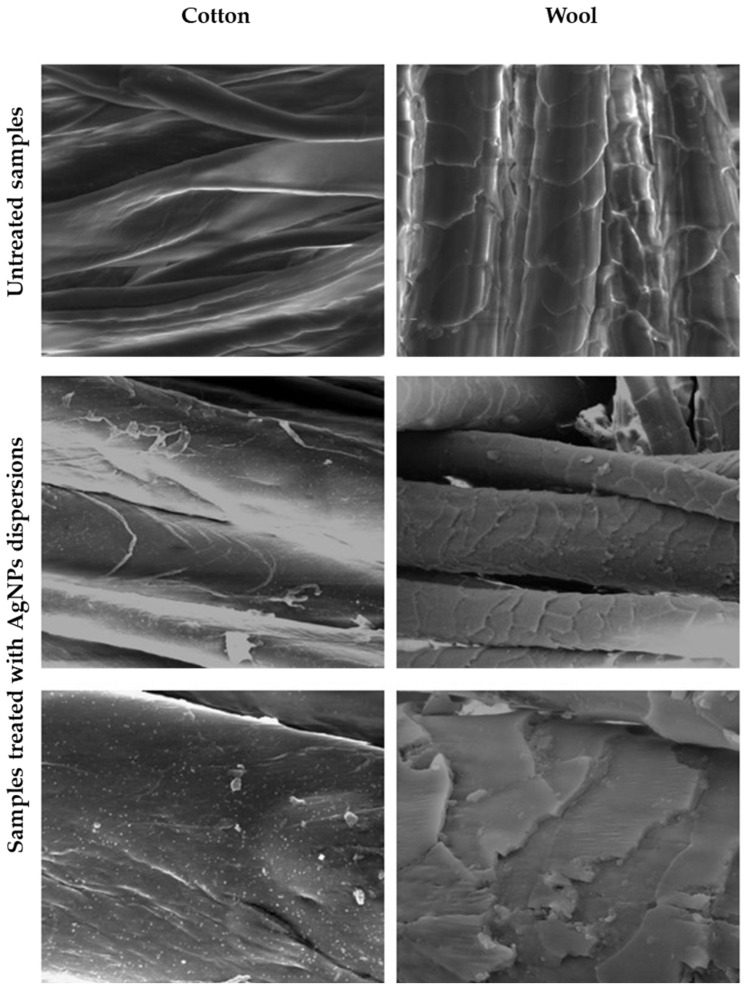
SEM images of cotton and wool textile samples, untreated and treated with AgNP dispersions, respectively.

**Figure 12:**
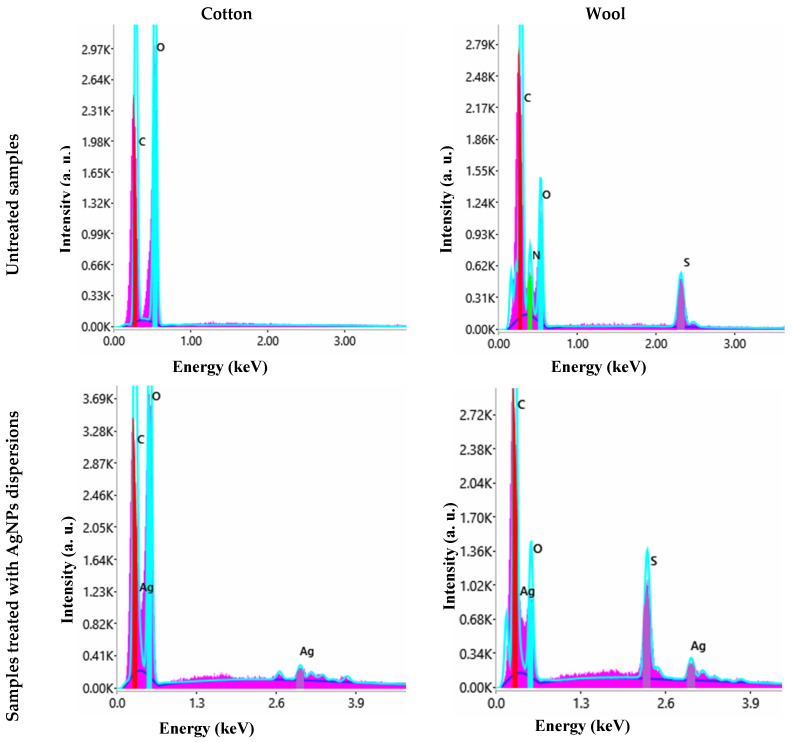
EDX spectra of untreated and treated cotton and wool samples with AgNP dispersions, respectively.

The efficiency of the treatment was first assessed by evaluating the morphology of the textile fibres before and after the application of the AgNPs dispersion (Figure 11), observing that the silver nanoparticles adhered uniformly to their surface. EDX spectra confirmed their nature and the presence of AgNPs (Figure 12).

### 3.7. Studying the Impact of AgNP Dispersions Synthesized Based on Crataegus Monogyna Extract on Textile Samples by Monitoring Chromatic Parameters

Table 2 contains the L* a* b* parameters determined for the textile samples and reported to the untreated ones. The colour change of AgNPs-coated cotton to a darker, yellowish colour showed an increase in the positive *redness-green* index (a*) from −0.27 to 0.63 and a decrease in the lightness factor (L*) from 93.48 to 88.67. The wool sample exhibited similar behaviour, with a decrease in lightness (L*) from 83.96 to 79.11. These changes are related to the deposition of the synthesized AgNPs on the cotton and wool surface [34,72], which is also confirmed by SEM and XRD analysis.

**Figure 13:**
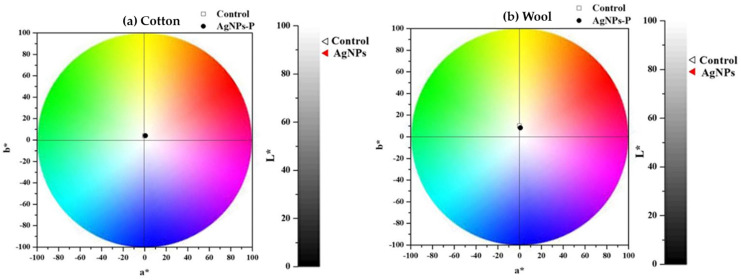
Chromatic diagrams of (**a**) cotton and (**b**) wool samples treated with AgNP dispersions, compared to untreated samples.

The total colour difference, ΔE*, was 4.89 for cotton and 5.24 for wool. Colour diagrams, illustrated in Figure 13, demonstrate that following the application of the AgNPs dispersion obtained with *Crataegus monogyna* extract on textile samples, their colour in both the case of cotton and wool did not undergo significant changes.

Unlike the other extracts studied, whose AgNP dispersions produced slight colour changes on cotton samples, the AgNP dispersion based on *Crataegus monogyna* extract can be used on both types of textiles.

### 3.8. Evaluation of Antimicrobial Activity

**Figure 14:**
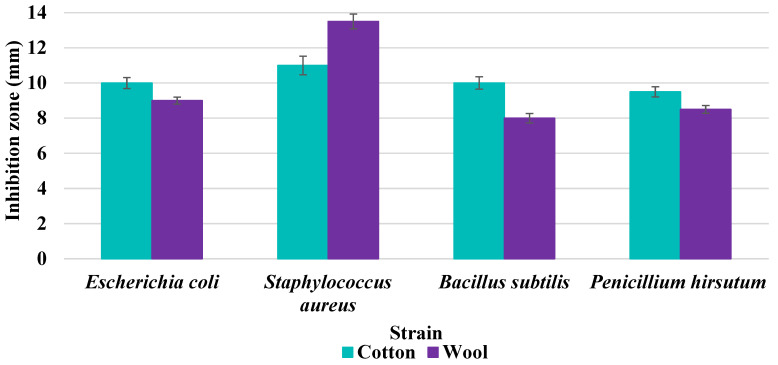
Dimensions of the inhibition zone formed on Petri plates inoculated with microbial strains and incubated with textile samples treated with AgNPs.

Antimicrobial properties were studied by evaluating the % bacterial reduction visualized by counting the colony-forming units (CFU) of the strains tested for both types of textiles (Table 3), which was 99.99%. These percentages demonstrate the bactericidal efficiency of the AgNP dispersion obtained with *Crataegus monogyna* extract. The images of the Petri dishes inoculated with the four tested microbial strains are presented in Table 4. They highlight the formation of clear zones of microbial inhibition around the cotton and wool samples, after treatment with AgNP. The sizes of the inhibition zones are compared in Figure 14.

For both cotton and wool textile samples, the size of the microbial inhibition zones is superior when *Crataegus monogyna* extract was used, compared to other extracts studied [55,65], with values between 8 and 13.5 mm. The considerable enhancement can be directly attributed to the interaction of available AgNPs towards the membranes of Gram-positive and Gram-negative bacteria.

AgNPs showed superior efficacy against bacterial strains compared to fungal strains. This behaviour demonstrates that direct contact between the AgNPs and the bacterial cell results in damage to cell walls and membranes, leading to cell death [73]. Thus, in the case of inhibition of *Staphylococcus aureus* bacteria, the best results are obtained for both cotton and wool.

These values were comparable with those obtained when using *Ageratum conyzoides* in the green AgNPs synthesis, for activated carbon compositing to prepare antimicrobial cotton fabric [74]. Moreover, there is no significant difference in bactericidal performance between gram-positive and gram-negative bacteria, compared to other studies. For example, when using *Citrus sinensis* fruit peel extract for producing AgNPs, the antibacterial efficiency of the material treated with the resulted dispersion is higher against gram- positive bacteria *S. aureus*, compared to *E. coli* [75]. Finally, it is worth noting that both antibacterial and antifungal activities are equally exhibited.

## 4. Conclusions

AgNPs with main diameters of 62 to 85 nm, spherical in shape, stable, and well-dispersed, were produced using different ratios of *Crataegus monogyna* extract: AgNO_3_. *Crataegus monogyna* extract used for silver ion reduction showed a relatively high phenolic content of 30.58 ± 2.20 mg/g dry plant, and a strong antioxidant activity with a Trolox equivalent of 96 ± 1.6 μmol/g dry plant. The polyphenol consumption was 1.58 mg, representing 5% of the total polyphenol content. The AgNPs dispersions exhibited superior antioxidant activity, short-lived free radical scavenging activity in the range 76–93% and long-lived free radical scavenging activity between 25–95%, which was influenced by the extract:AgNO_3_ ratio. A strong argument responsible for the efficiency of capturing ABTS^●+^ radicals is correlated with the higher content of *Crataegus monogyna* extract. On the other hand, when the extract content is lower, the increase in antioxidant activity is due to the nanosized effect of AgNPs.

Several cotton and wool samples treated with AgNPs were morphologically, chromatically and antimicrobial evaluated. SEM images showed that AgNPs adhered uniformly to the textile fibre surface. The chromatic effect of these metal nanoparticles on textile materials was minor (ΔE* was 4.89 for cotton and 5.24 for wool), and the antimicrobial activity against *Escherichia coli*, *Staphylococcus aureus*, *Bacillus subtilis*, as well as the fungal strain of *Penicillium hirsutum*, was superior. For both cotton and wool textile samples, microbial inhibition was superior, i.e., 8–13.5 mm, using *Crataegus monogyna* extract, compared to other reported extracts. The considerable improvement detected can be attributed to the ability of AgNPs to interact and disrupt the permeability of the Gram-positive and Gram-negative cell membranes. As a result of these cumulative results, it can be confidently stated that obtaining these AgNPs with the involvement of *Crataegus monogyna* extract represents an effective and suitable antimicrobial treatment for both types of textile samples, both cotton and wool.

## Figures and Tables

**Table 1:** Compounds from *Crataegus monogyna* extract identified by FT-ICR MS.

Compound	Molecular Formula	Calculated Mass (m/z)		Measured Mass (m/z)		Mass Accuracy (ppm)	
		ESI+	ESI−	ESI+	ESI−	ESI+	ESI−
Epicatechin	C_15_H_14_O_6_	291.086315	289.071762	291.086179	289.071621	−0.467215	−0.487768
Quercetin	C_15_H_10_O_7_	303.049929	-	303.049831	-	−0.323379	-
Quercetin 3-O-rhamnoside-7-O-glucoside	C_27_H_30_O_16_	611.160661	609.146108	611.1600389	609.145543	−1.017899	−0.927528
3-Methylquercetin	C_16_H_12_O_7_	317.065579	-	317.065498	-	−0.255468	-
Quercetin-3-D-xyloside	C_20_H_18_O_11_	-	433.077635	-	433.077572	-	−0.145470
5-Desoxyquercetin	C_15_H_10_O_6_	287.055014	-	287.054990	-	−0.083608	-
Chlorogenic acid	C_16_H_18_O_9_	355.102359	353.087806	355.102272	353.087599	−0.245000	−0.586256
4,5-Dicaffeoylquinic acid	C_25_H_24_O_12_	517.134053	515.119500	517.133259	515.119135	−1.535385	−0.708573
Vitexin/Apigenin-7-O-glucoside	C_21_H_20_O_10_	433.112923	431.098370	433.112778	431.098090	−0.334786	−0.649504
2″-O-α-L-Rhamnopyranosyl-isovitexin	C_27_H_30_O_14_	579.170832	577.156279	579.170522	577.155674	−0.535248	−1.048243
Isoorientin/Luteolin-7-O-glucoside	C_21_H_20_O_11_	449.107838	447.093285	449.107728	447.092835	−0.244930	−1.006501
Hyperoside	C_21_H_20_O_12_	465.102753	463.088200	465.102584	463.087851	−0.363361	−0.753636
Rhoifolin (Apigenin 7-O-neohesperidoside)	C_27_H_30_O_14_	579.170832	-	579.170618	-	−0.369494	-
Apigenin 6,8-di-C-glucoside	C_27_H_30_O_15_	595.165747	593.151194	595.165468	593.149057	−0.468777	−3.602791
6-Arabinosyl-8-galactosylapigenin	C_26_H_28_O_14_	565.155182	563.140629	565.154995	563.140115	−0.330883	−0.912738
Isovitexin	C_21_H_20_O_10_	433.112923	-	433.112951	-	0.064648	-

**Table 2:** Chromatic parameters of textile samples (cotton and wool) treated with AgNP dispersions.

Sample		L*	a*	b*	∆L*	∆a*	∆b*	∆E*
**Untreated cotton**		93.48	−0.27	3.83	-	-	-	-
**Untreated** **wool**		83.96	−0.19	10.17	-	-	-	-
**AgNPs**	**Cotton**	88.67	0.63	3.99	−4.81	0.90	0.16	**4.89**
	**Wool**	79.11	0.60	8.35	−4.85	0.79	−1.82	**5.24**

**Table 3:** Antibacterial test results after a treatment of textiles with AgNPs.

Bacteria Strain	Textile Sample	CFUs/mL for Reference Samples	CFUs/mL for Samples Treated with AgNP Dispersion	Bacteria Reduction Percentage (%)
*Escherichia coli*	Cotton	2.1 × 10^4^	0	99.99
	Wool	2.8 × 10^4^	0	99.99
*Staphylococcus aureus*	Cotton	4.5 × 10^4^	0	99.99
	Wool	5.5 × 10^4^	0	99.99
*Bacillus subtilis*	Cotton	2.9 × 10^4^	0	99.99
	Wool	3.7 × 10^4^	0	99.99

**Table 4:** Images of Petri dishes inoculated with microbial strains and incubated with AgNP-treated textile samples.

Textile Sample	Cotton	Wool
Microbial Strain		
*Escherichia coli*	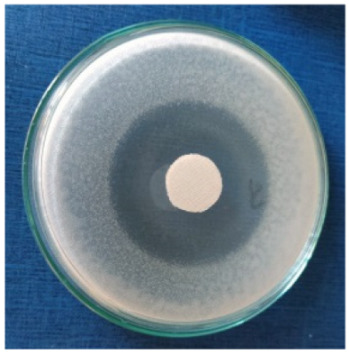	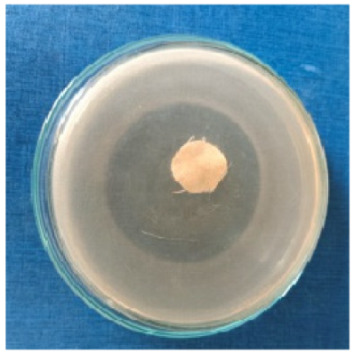
*Staphylococcus aureus*	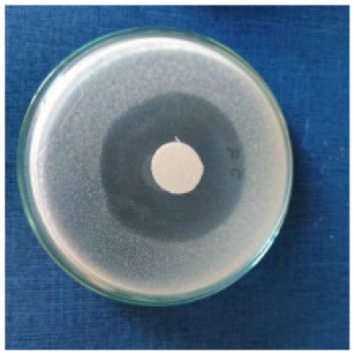	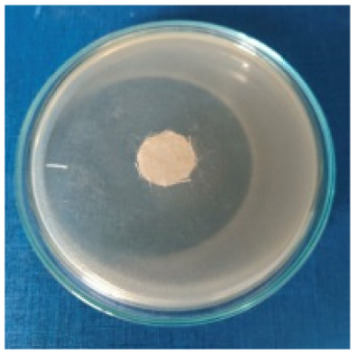
*Bacillus subtilis*	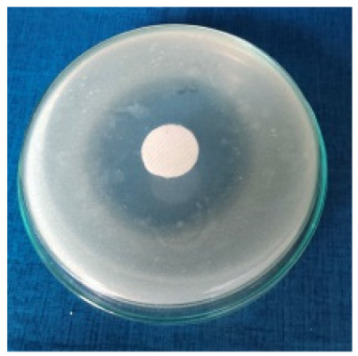	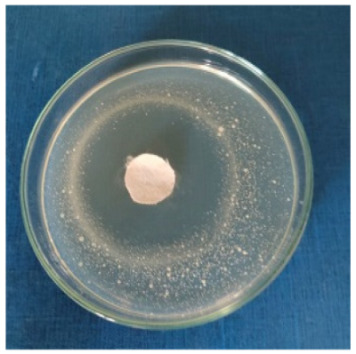
*Penicillium hirsutum*	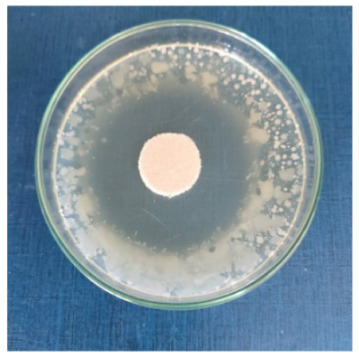	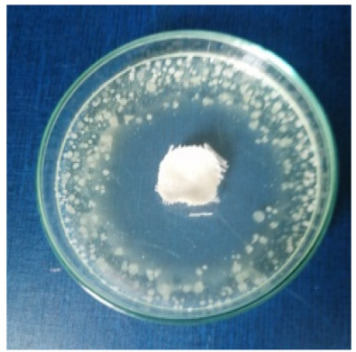

## Data Availability

The data are included in the text.

## Supplementary Materials

The following supporting information can be downloaded at: https://www.mdpi.com/article/10.3390/biomimetics10110737/s1. Figure S1. MS spectra of epicatechin (C_15_H_14_O_6_) obtained by negative ionization ESI− (m/z = 289.0716) and positive ionization ESI+ (m/z = 291.0861). Figure S2. MS spectrum of quercetin (C_15_H_10_O_7_) obtained by positive ionization ESI+ (m/z = 433.0776). Figure S3. MS spectrum of 5-deoxy-quercetin (C_15_H_10_O_6_) obtained by positive ionization ESI+ (m/z = 287.0549). Figure S4. MS spectra of 3-O-rhamnoside-7-O-glucoside-quercetin (C_27_H_30_O_16_) obtained by negative ionization ESI− (m/z = 609.1455) and positive ionization ESI+ (m/z = 611.1600). Figure S5. MS spectrum of 3-methylquercetin (C_16_H_12_O_7_) obtained by positive ionization ESI+ (m/z = 317.0655). Figure S6. MS spectrum of 3-D-xyloside-quercetin (C_20_H_18_O_11_) obtained by negative ionization ESI− (m/z = 433.0775). Figure S7. MS spectra of chlorogenic acid (C_16_H_18_O_9_) obtained by negative ionization ESI− (m/z = 353.0876) and positive ionization ESI+ (m/z = 355.1022). Figure S8. MS spectra of 4,5-dicaffeoylquinic acid (C_25_H_24_O_12_) obtained by negative ionization ESI− (m/z = 515.1191) and positive ionization ESI+ (m/z = 517.1332). Figure S9. MS spectra of 7-O-glucoside-apignene (vitexin) (C_21_H_20_O_10_) obtained by negative ionization ESI− (m/z = 431.0980) and positive ionization ESI+ (m/z = 433.1127). Figure S10. MS spectrum of 3-D-xyloside-quercetin (C_20_H_18_O_11_) obtained by negative ionization ESI− (m/z = 433.0775). Figure S11. MS spectrum of 5-deoxyquercetin (C_15_H_10_O_6_) obtained by positive ionization ESI+ (m/z = 287.0549). Figure S12. MS spectra of 2″-O-α-L-rhamnopyranosyl-isovitexin (C_27_H_30_O_14_) obtained by negative ionization ESI− (m/z = 577.1556) and positive ionization ESI+ (m/z = 579.1705). Figure S13. MS spectra of 7-O-glucoside-luteolin (isoorientin) (C_21_H_20_O_11_) obtained by negative ionization ESI− (m/z = 447.0928) and positive ionization ESI+ (m/z = 449.1077). Figure S14. MS spectra of hyperoside (C_21_H_20_O_12_) obtained by negative ionization ESI− (m/z = 463.0878) and positive ionization ESI+ (m/z = 465.1025).
