# Supplementary material for: Antimicrobial Efficacy of Green Silver Nanoparticles Synthesized Using Crataegus monogyna Extract
Source: Biomimetics (Basel). 2025 Nov 3;10(11):737. doi: 10.3390/biomimetics10110737 (PMC12649965; doi:10.3390/biomimetics10110737)
Supplement: Supplementary file 1 [file biomimetics-10-00737-s001.zip › Supplementary Material.pdf]

Supplementary Material (Figures S1–S14): FT-MS characterization of *Crataegus monogyna* extract

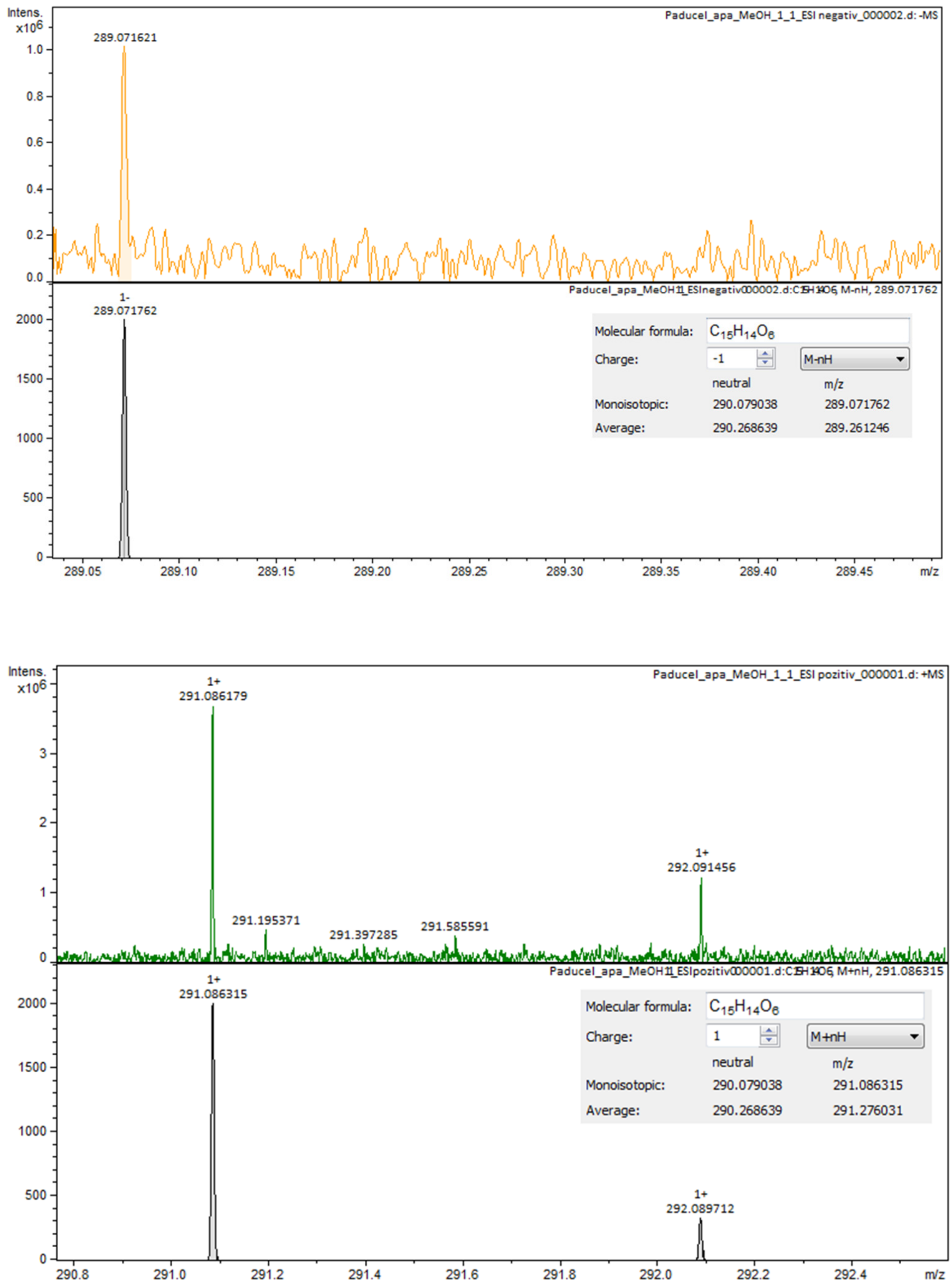

Figure S1. MS spectra of epicatechin ($C_{15}H_{14}O_6$) obtained by negative ionization ESI- (m/z =289.0716) and positive ionization ESI+ (m/z = 291.0861).

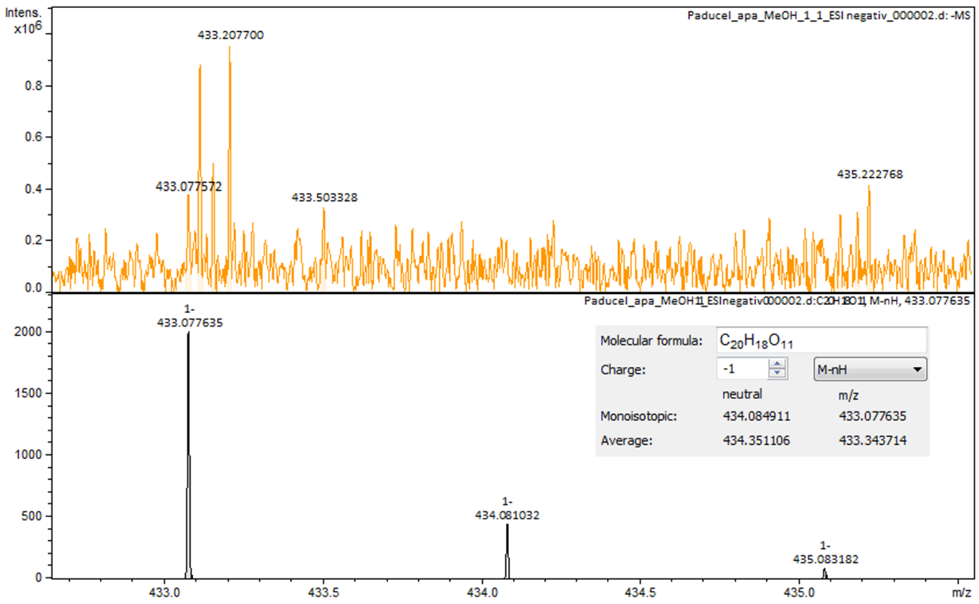

Figure S2. MS spectrum of quercetin ($C_{15}H_{10}O_7$) obtained by positive ionization ESI+ (m/z = 433.0776).

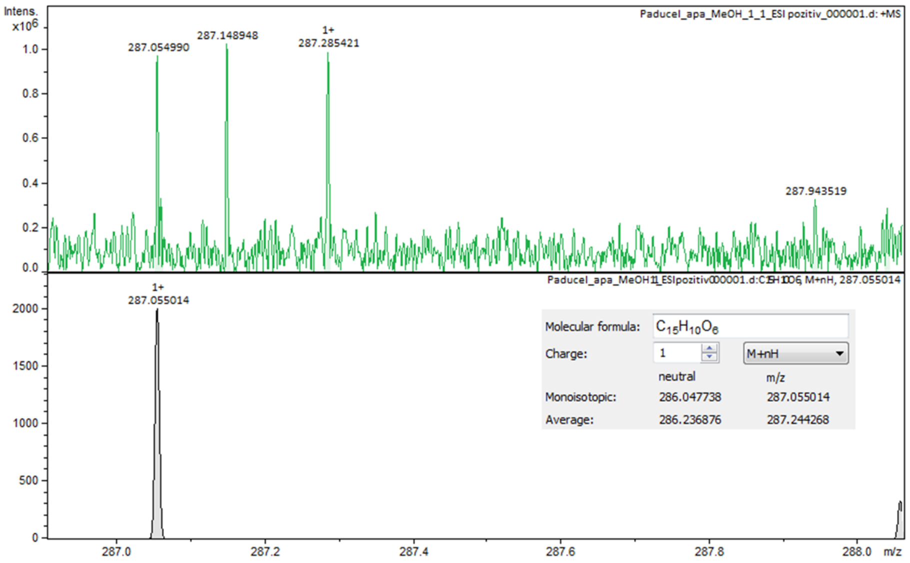

Figure S3. MS spectrum of 5-deoxy-quercetin ($C_{15}H_{10}O_6$) obtained by positive ionization ESI+ (m/z = 287.0549).

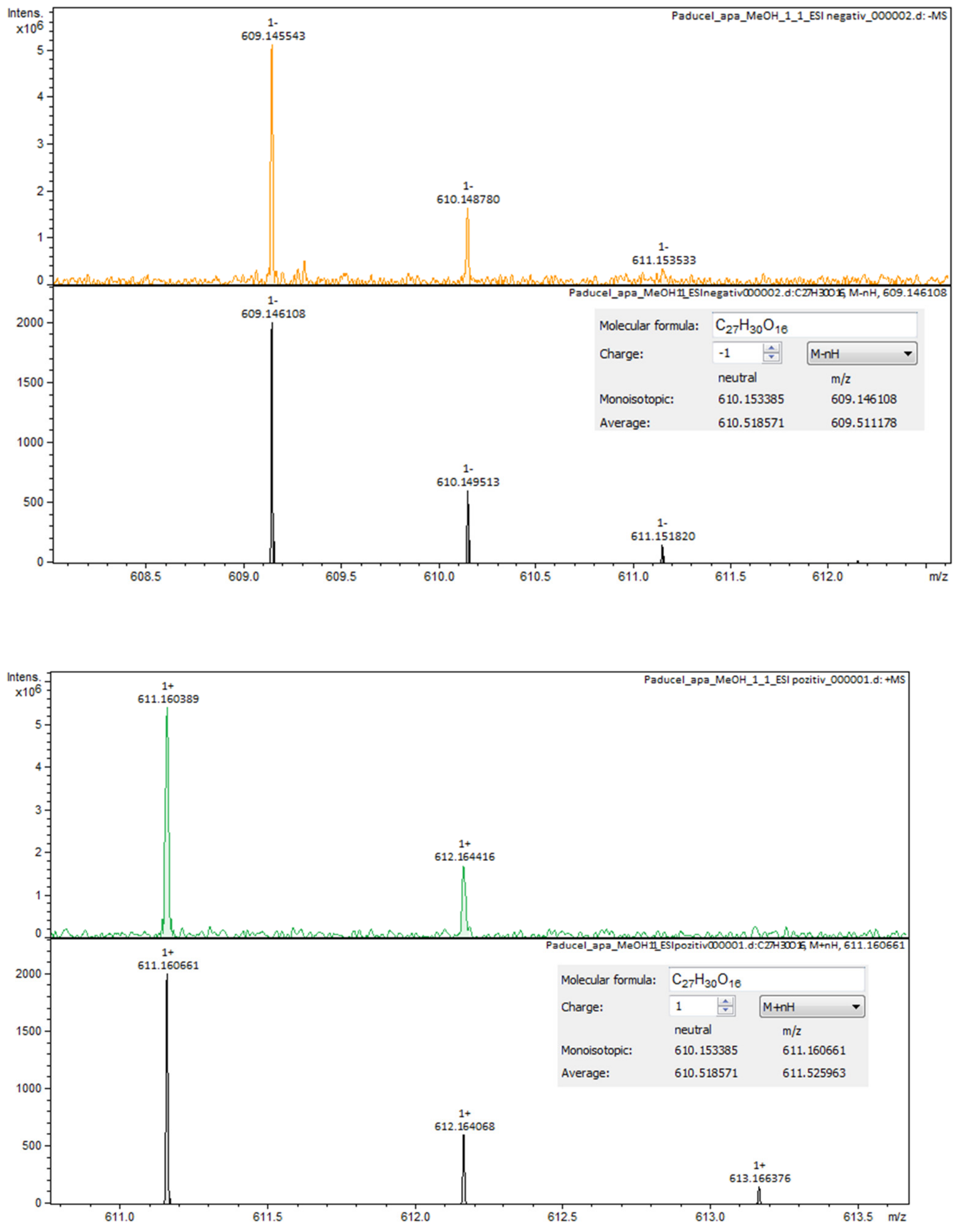

Figure S4. MS spectra of 3-O-rhamnoside-7-O-glucoside-quercetin ($C_{27}H_{30}O_{16}$) obtained by negative ionization ESI- (m/z =609.1455) and positive ionization ESI+ (m/z = 611.1600).

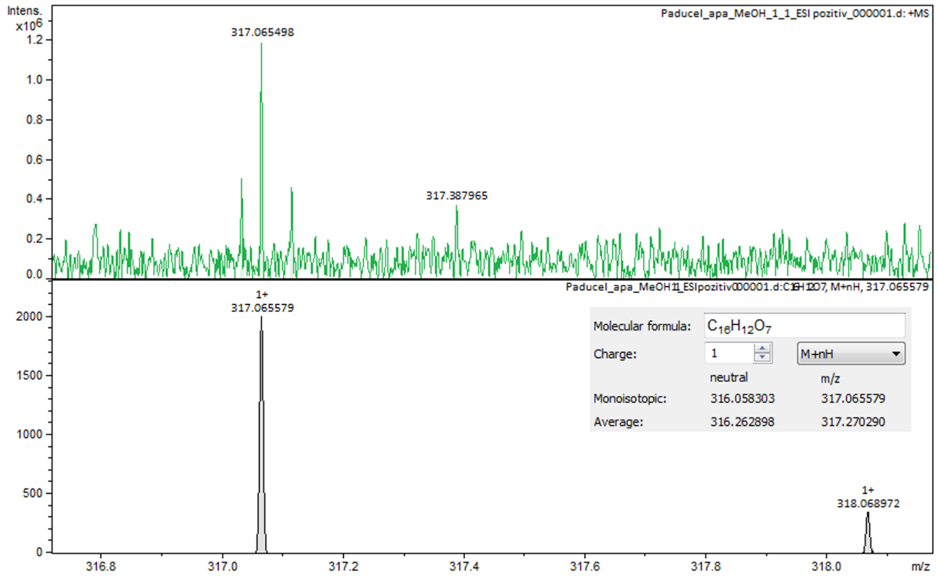

Figure S5. MS spectrum of 3-methylquercetin ($C_{16}H_{12}O_7$) obtained by positive ionization ESI+ (m/z = 317.0655).

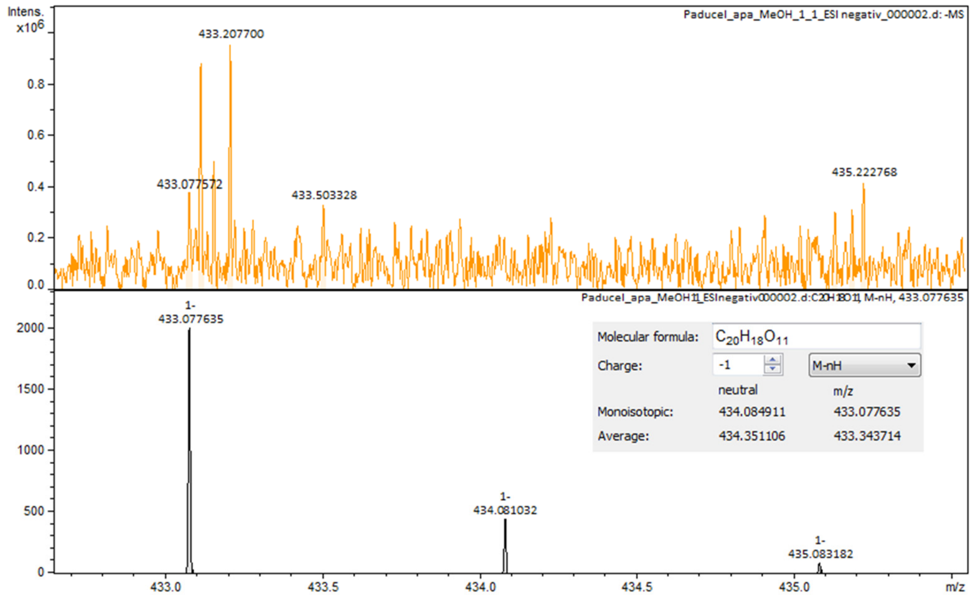

Figure S6. MS spectrum of 3-D-xyloside-quercetin (C20H18O11) obtained by negative ionization ESI- (m/z = 433.0775).

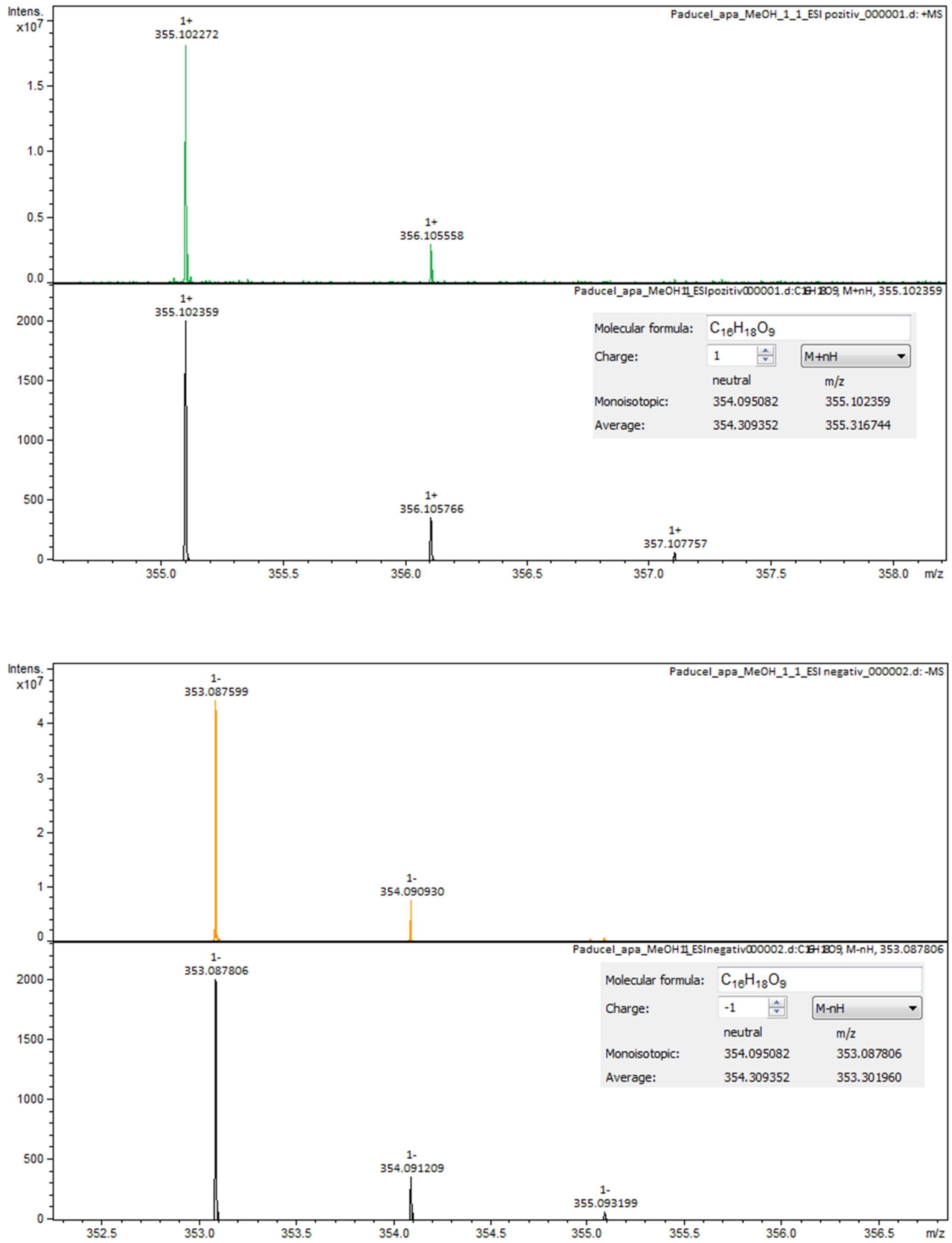

Figure S7. MS spectra of chlorogenic acid ($C_{16}H_{18}O_9$) obtained by negative ionization ESI- (m/z =353.0876) and positive ionization ESI+ (m/z = 355.1022).

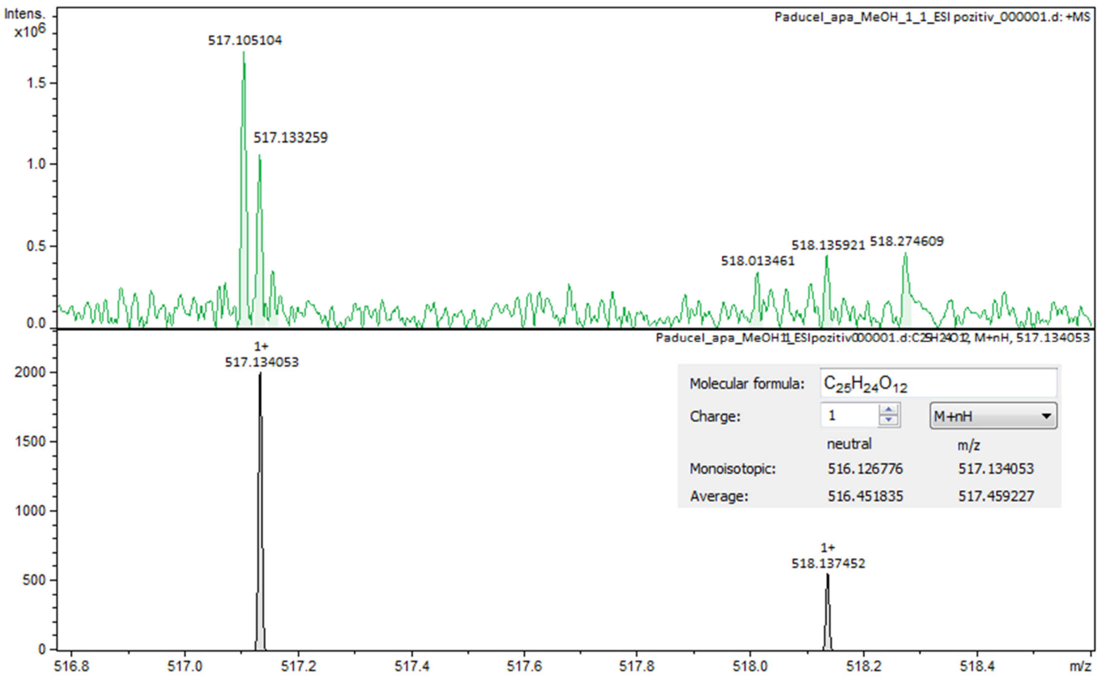

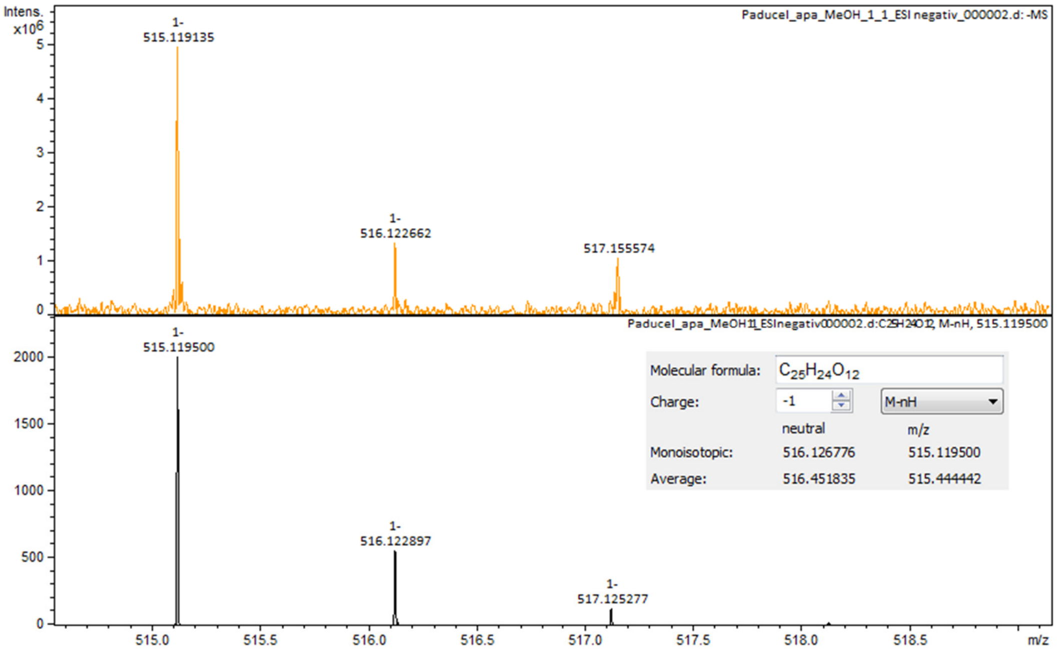

Figure S8. MS spectra of 4,5-dicaffeoylquinic acid ($C_{25}H_{24}O_{12}$) obtained by negative ionization ESI- (m/z =515.1191) and positive ionization ESI+ (m/z = 517.1332).

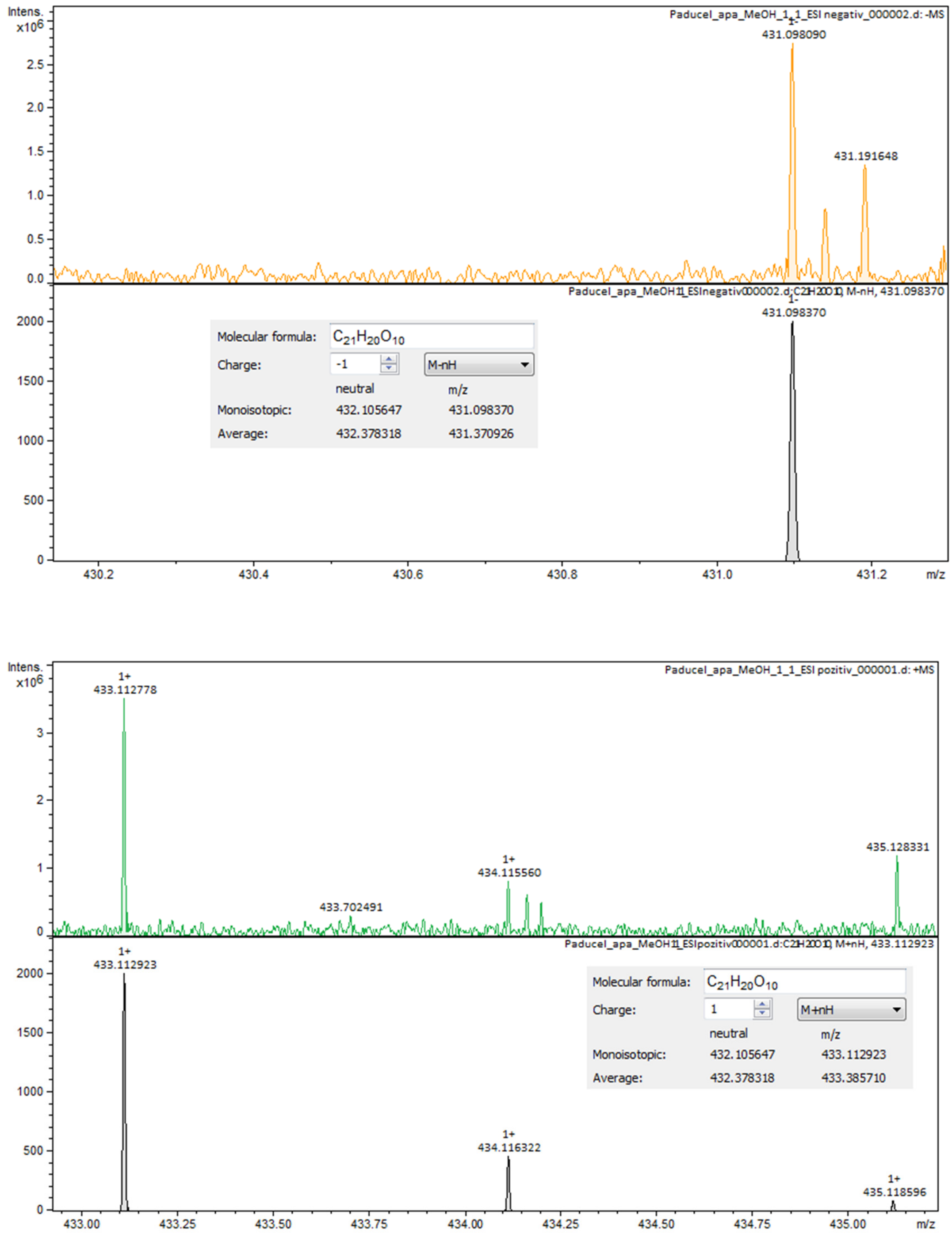

Figure S9. MS spectra of 7-O-glucoside-apignene (vitexin) ($C_{21}H_{20}O_{10}$) obtained by negative ionization ESI- (m/z =431.0980) and positive ionization ESI+ (m/z = 433.1127).

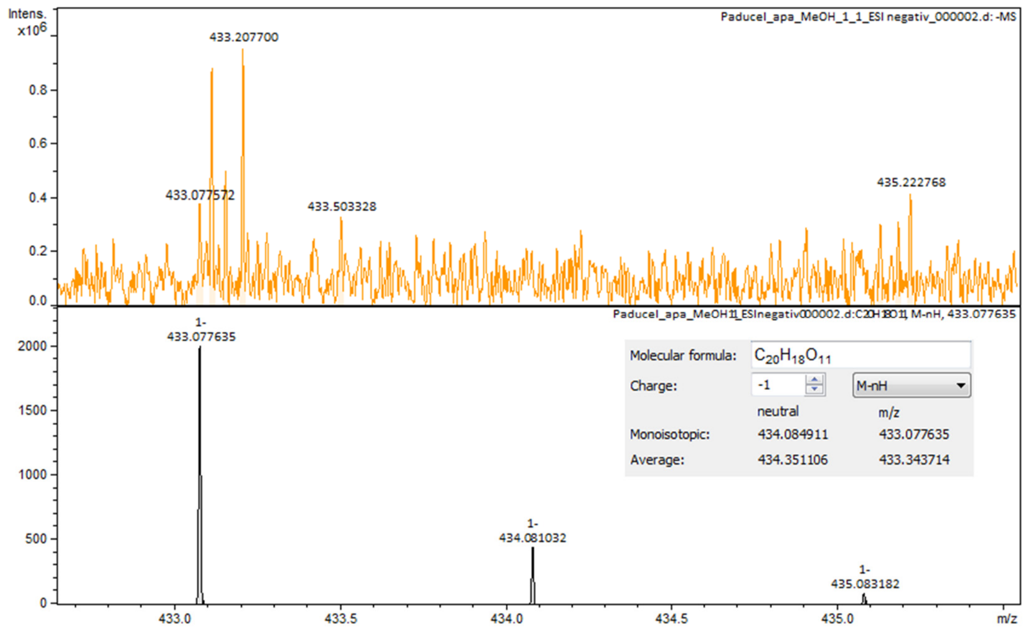

Figure S10 MS spectrum of 3-D-xyloside-quercetin ($C_{20}H_{18}O_{11}$) obtained by negative ionization ESI- (m/z = 433.0775).

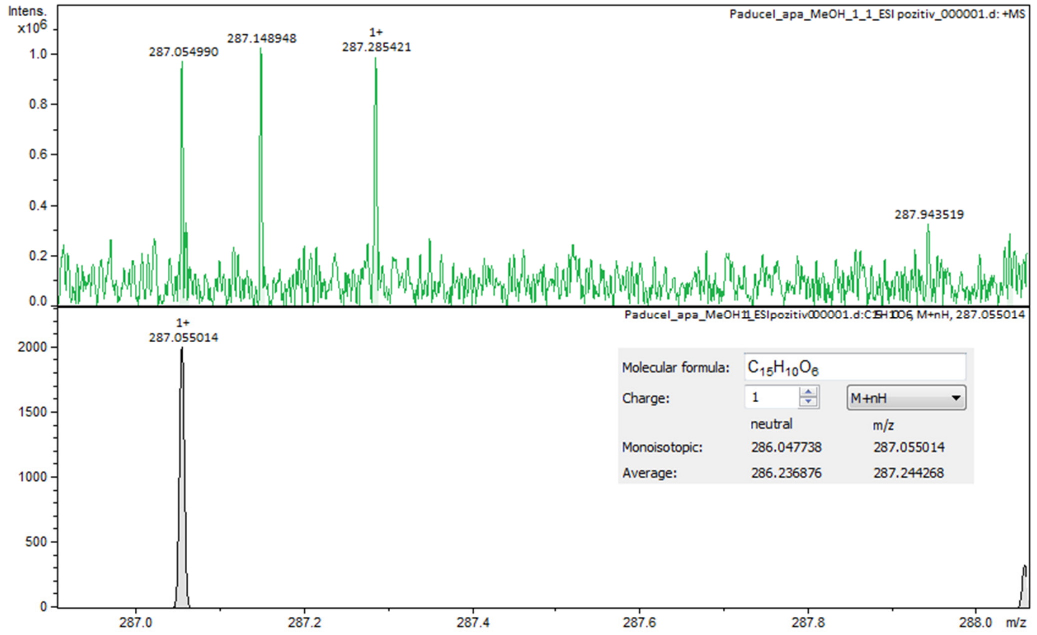

Figure S11. MS spectrum of 5-deoxyquercetin ($C_{15}H_{10}O_6$) obtained by positive ionization ESI+ (m/z = 287.0549).

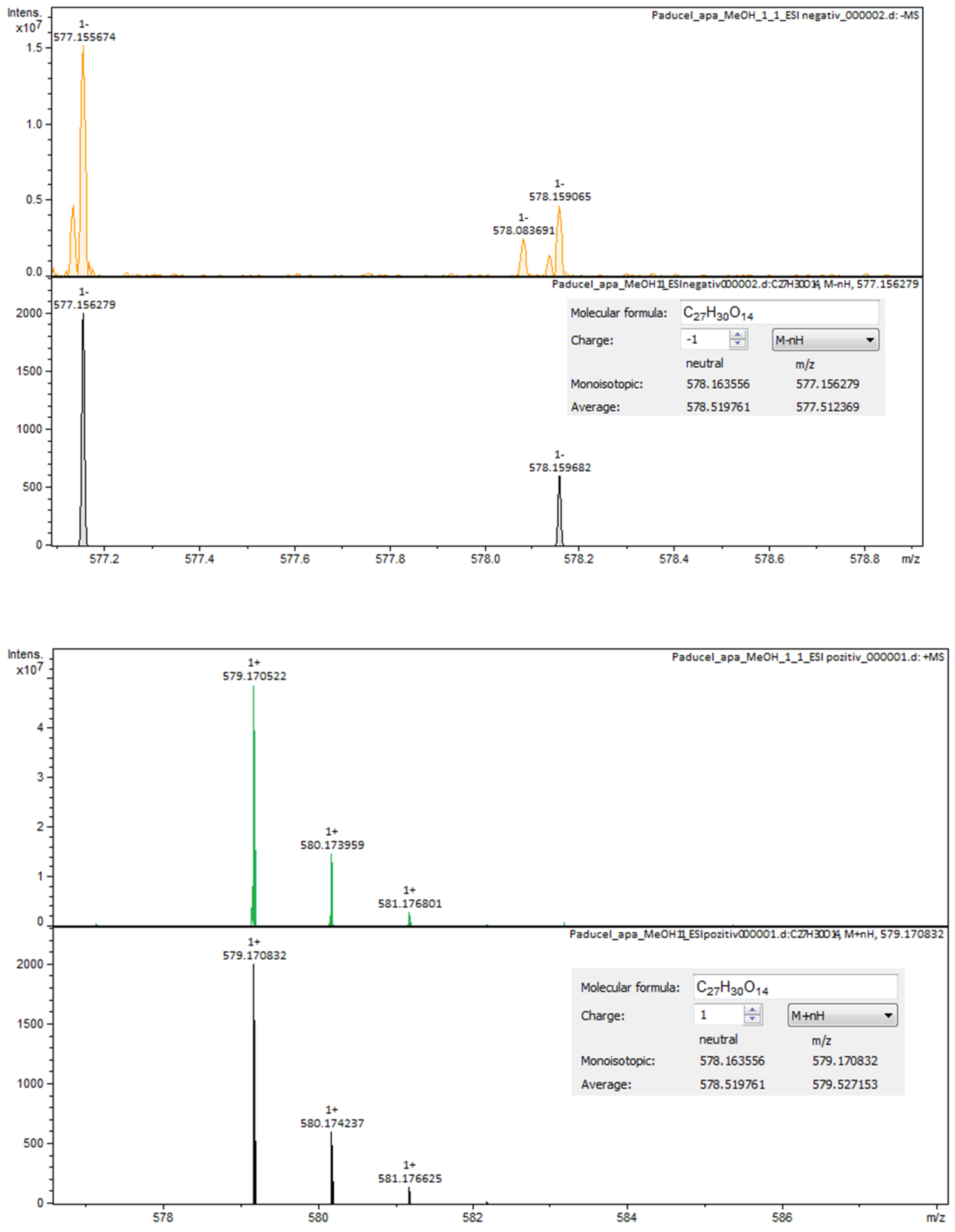

Figure S12. MS spectra of 2″-O-α-L-rhamnopyranosyl-isovitexin ($C_{27}H_{30}O_{14}$) obtained by negative ionization ESI- (m/z =577.1556) and positive ionization ESI+ (m/z = 579.1705).

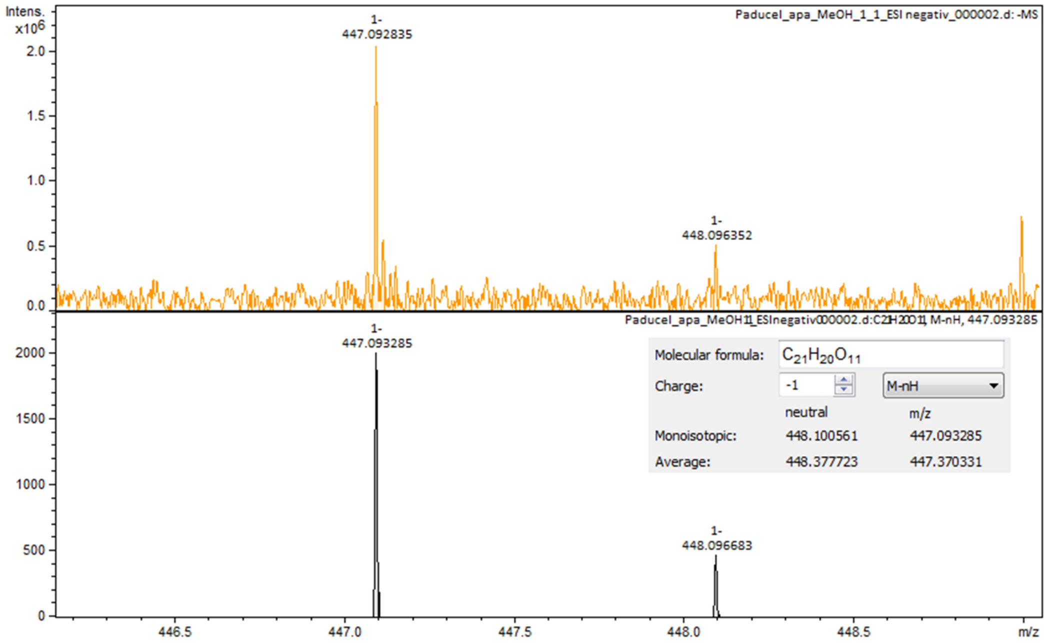

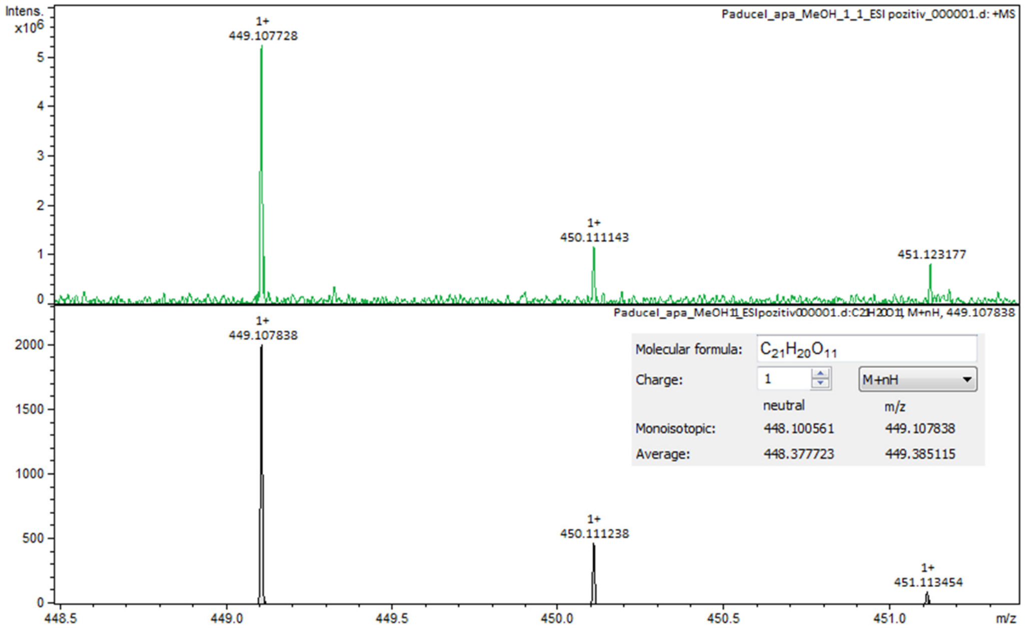

Figure S13. MS spectra of 7-O-glucoside-luteolin (isoorientin) ($C_{21}H_{20}O_{11}$) obtained by negative ionization ESI- (m/z =447.0928) and positive ionization ESI+ (m/z = 449.1077).

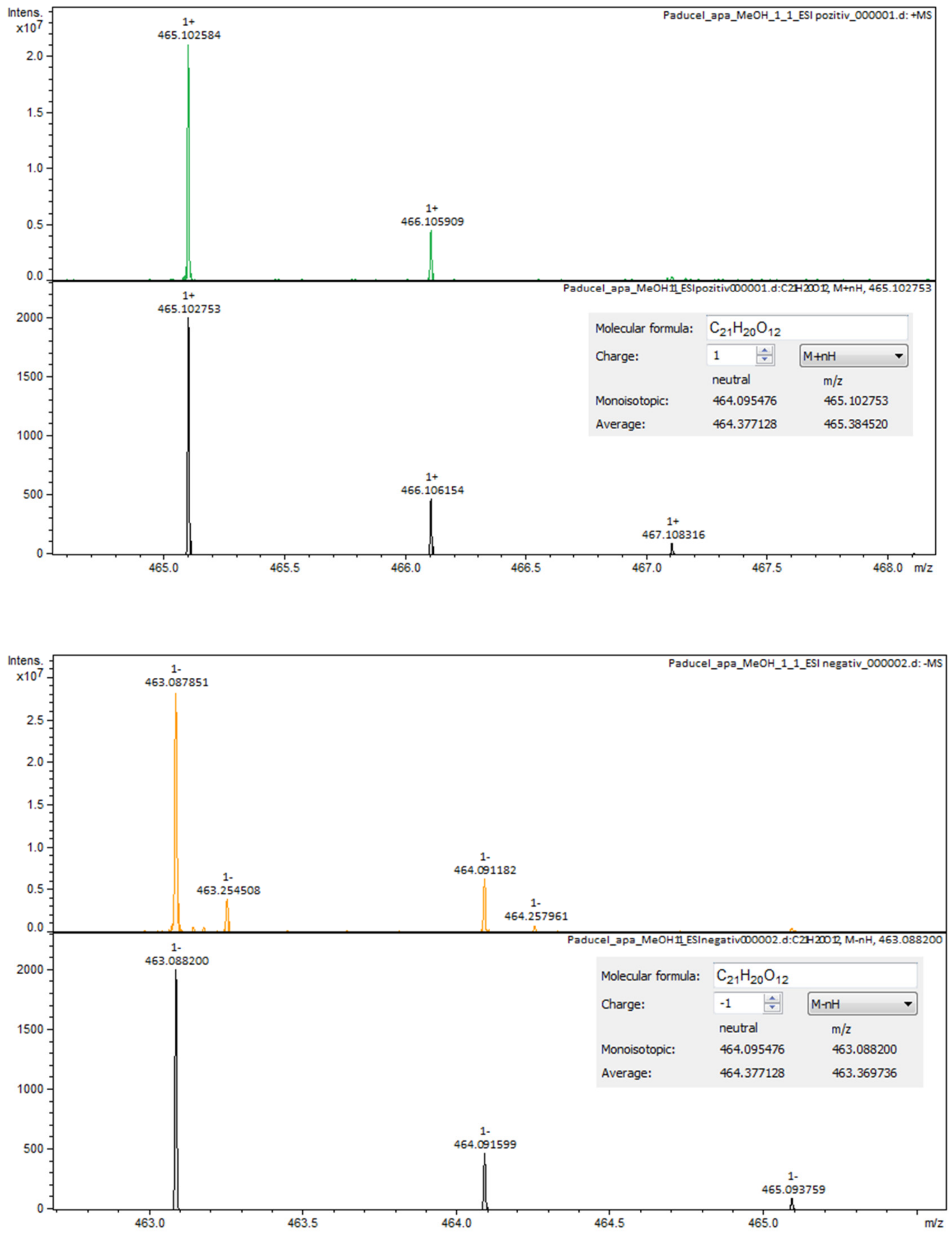

Figure S14. MS spectra of hyperoside ($C_{21}H_{20}O_{12}$) obtained by negative ionization ESI- (m/z =463.0878) and positive ionization ESI+ (m/z = 465.1025).
